# Acute exercise‐induced lipolysis and increase in levels of fatty acid esters of hydroxy fatty acids in adipose tissue are impaired in obese and aged mice

**DOI:** 10.1113/EP093561

**Published:** 2026-07-25

**Authors:** Marko Mitrovic, Olga Horakova, Martin Riecan, Veronika Kleinova, Tomas Cajka, Lenka Rossmeislova, Ondrej Kuda, Martin Rossmeisl

**Affiliations:** ^1^ Laboratory of Adipose Tissue Biology Institute of Physiology of the Czech Academy of Sciences Prague Czech Republic; ^2^ First Faculty of Medicine Charles University Prague Czech Republic; ^3^ Laboratory of Metabolism of Bioactive Lipids Institute of Physiology of the Czech Academy of Sciences Prague Czech Republic; ^4^ Laboratory of Translational Metabolism Institute of Physiology of the Czech Academy of Sciences Prague Czech Republic; ^5^ Department of Pathophysiology, Center for Research on Nutrition, Metabolism and Diabetes, Third Faculty of Medicine Charles University Prague Czech Republic

**Keywords:** acute exercise, adipose tissue, fatty acid esters of hydroxy fatty acids

## Abstract

Improving adipose tissue (AT) metabolism through exercise might increase production of lipokines from the fatty acid esters of hydroxy fatty acids (FAHFA) family. Although exercise training can increase insulin sensitivity and levels of specific FAHFA in AT and the circulation in humans, the effects of acute exercise on FAHFA levels remain unclear. Previously untrained lean, obese and aged male C57BL/6J mice were subjected to acute treadmill exercise. AT lipolysis, metabolomic profile and FAHFA levels were analysed at baseline and 0 and 3 h postexercise. Baseline AT levels of FAHFA, particularly those containing mono‐ and polyunsaturated fatty acids, were lower in obese and aged mice compared with lean mice. AT‐specific expression of genes related to *de novo* lipogenesis and lipolysis was reduced, particularly in obese mice, whereas expression of FAHFA hydrolysis‐related genes was higher in aged mice. In lean mice, acute exercise increased plasma non‐esterified fatty acids and their release from AT explants, which was suppressed in obese and aged counterparts. Only in lean animals, acute exercise led to a time‐dependent increase in total FAHFA levels in AT, with the most pronounced increase observed in 13/12‐regioisomers from different FAHFA subfamilies. The effect of acute exercise on AT FAHFA levels was stronger than that of fasting alone, revealing palmitoleic acid‐containing FAHFA as the most differentiating regioisomers. In contrast, plasma FAHFA levels were minimally affected in all groups. In conclusion, acute exercise increases FAHFA levels in AT but not in plasma, and this effect appears to be negatively affected by obesity and ageing.

## Abstract (highlights)

- **What is the central question of this study?**
  The study investigates whether acute exercise affects levels of fatty acid esters of hydroxy fatty acids (FAHFA) in adipose tissue and the bloodstream and how these effects change with obesity and ageing.
- **What is the main finding and its importance?**
  Obese and aged mice had lower baseline adipose tissue FAHFA. Exercise increased adipose tissue lipolysis and FAHFA levels in lean mice, but this effect was blunted in obese and aged animals. Thus, obesity and ageing not only alter baseline adipose tissue FAHFA but also impair FAHFA regulation in response to acute metabolic stress.

## 1 INTRODUCTION

Physical activity has many health benefits (Ruegsegger & Booth, 2018), largely based on altering the secretory activity of various tissues (Chow et al., 2022). Most research to date has focused on the effects of exercise on skeletal muscle myokines (Severinsen & Pedersen, 2020). However, emerging evidence suggests that improving the function of non‐contractile tissues, such as adipose tissue (AT), might contribute to the metabolic benefits of exercise (Chow et al., 2022; Stanford & Goodyear, 2016). There is a bidirectional interaction between AT and systemic metabolism, which is influenced by various (epi)genetic, environmental and lifestyle factors, with AT‐secreted products (i.e., adipokines) and other bioactive substances helping to regulate energy balance and insulin sensitivity (Li et al., 2020; Santoro & Kahn, 2023).

Adipose tissue has been described as a major source of a new class of lipids (i.e., lipokines) called fatty acid esters of hydroxy fatty acids (FAHFA) (Yore et al., 2014). The evidence that some FAHFA species, including palmitic acid esters of hydroxy stearic acid (PAHSA), have potent anti‐inflammatory and anti‐diabetic properties (Aryal et al., 2021; Riecan et al., 2022; Yore et al., 2014; Zhou et al., 2019) has sparked interest in investigating their metabolism further. It has been demonstrated that levels of various FAHFA are lower in pathophysiological conditions associated with AT dysfunction (Hammarstedt et al., 2018; Yore et al., 2014) and are related to body mass index, age and diet (Kellerer et al., 2021). Besides PAHSA, which represent the most studied group of FAHFA (Riecan et al., 2022), identification of other FAHFA families and regioisomers continues, raising questions about their characteristics and primary biological functions. For instance, unsaturated FAHFA with branching points further away from the carboxyl group are more likely to enhance glucose‐stimulated insulin secretion, whereas those with branching points closer to the carboxyl group are more likely to be anti‐inflammatory (Aryal et al., 2021). In many cases, the biological properties, regulatory mechanisms and therapeutic potential of FAHFA remain unknown. This also applies to physical activity and exercise, where only a limited number of FAHFA‐related studies exist (Brezinova et al., 2020; Nelson et al., 2022).

We have recently demonstrated that exercise training for 4 months increased serum and AT PAHSA levels in older sedentary women (Brezinova et al., 2020). This suggests that chronic exercise might stimulate not only the synthesis of PAHSA in AT but also their release into the circulation. Indeed, adipose triglyceride lipase (ATGL), which has been identified as a biosynthetic enzyme for FAHFA in addition to its role in triacylglycerol (TG) lipolysis (Brejchova et al., 2021; Patel et al., 2022), is activated by exercise in AT together with hormone‐sensitive lipase (HSL) (Laurens et al., 2020; Muscella et al., 2020). Repeated activation of ATGL during each acute exercise bout might thus contribute to the stimulatory effects of chronic exercise on FAHFA levels in AT. Additionally, a single bout of exercise is associated with oxidative stress and an inflammatory response (Ji et al., 2006), processes known to be involved in FAHFA synthesis in AT (Ertunc et al., 2024; Kuda et al., 2018; Paluchova et al., 2022). However, little is known about how AT and circulating FAHFA levels are regulated in response to acute exercise in untrained individuals and how this is affected by obesity or age.

The aim of this study was to investigate the effect of a single bout of exercise on the lipolytic response and FAHFA levels in lean, obese and aged mice, using AT and plasma lipidomic profiling.

## 2 MATERIALS AND METHODS

### 2.1 Ethical approval

Animal experiments were approved by the Institutional Animal Care and Use Committee and the Committee for Animal Protection of the Czech Academy of Sciences (approval number: 62/2023), following the EU Directive 2010/63/EU on protecting animals used for scientific purposes.

### 2.2 Animals and diets

Male C57BL/6J mice (Charles River Laboratories, Sulzfeld, Germany) were obtained at an age of 12 weeks (lean group) or 8 weeks (obese group), whereas the aged mice (∼15‐month‐old) were obtained from The Jackson Laboratory (Bar Harbor, ME, USA). Mice were housed individually and maintained at ∼22°C on a 12 h–12 h light–dark cycle, with ad libitum access to water and standard chow (‘Rat/Mouse‐Maintenance extrudate’; ssniff Spezialdiäten GmbH; Soest, Germany). After 2 weeks of adaptation, mice assigned to the obese group were switched to a lard‐based high‐fat diet (HFD; ∼21 kJ/g; product ‘DIO‐60 kJ% fat (Lard)’; ssniff Spezialdiäten GmbH) for 11 weeks. Mice in the lean group received standard chow for ∼7 weeks prior to the experiment.

### 2.3 Experimental set‐up and exercise protocol

At the time of the acute exercise intervention, the lean, obese and aged mice were 4.5, 5 and 18 months old, respectively (Table 1). The design of the acute exercise protocol emerged from several pilot experiments (Figure A1) aimed at achieving maximal stimulation of AT lipolysis. A six‐lane treadmill (Exer‐3/6; Columbus Instruments; Columbus, OH, USA) was used for the exercise. On the day of the experiment, mice were deprived of food between 11:00 and 15:00 h, then subjected to the following protocol: 8 m/min at 5% incline for 40 min, followed by an increase of 0.5 m/min every 10 min until exhaustion. Exercising mice (*n *= 6) from both lean and obese groups were killed either immediately after exercise (0 min) or 3 h postexercise, whereas aged mice were analysed only at the 0 min time point. Separate groups of mice (*n *= 5 or 6) within all experimental cohorts were killed at 15:00 h (after 4 h without food) to obtain baseline values. Control groups were used for each time point (*n *= 4 or 5; same manipulation, but without running).

**TABLE 1:** Basal parameters in lean, obese and aged C57BL/6J mice.

Parameter	Lean	Obese	Aged
Body weight, g	29.2 ± 1.6	45.0 ± 5.4^*^	42.8 ± 9.3^*^
Age, months	4.5	5	18
**Adiposity**			
Epididymal AT, g	0.48 ± 0.14	2.64 ± 0.54^*^	1.16 ± 0.37^*^, ^#^
Subcutaneous AT, g	0.15 ± 0.03	0.90 ± 0.26^*^	0.52 ± 0.47^*^, ^#^
Adiposity index, % body weight	2.13 ± 0.40	7.81 ± 0.90^*^	3.76 ± 0.97^*^, ^#^
**Liver parameters**			
Weight, g	1.21 ± 0.17	1.54 ± 0.31	1.95 ± 0.79
Glycogen content, mg/g	9.3 ± 2.9	8.2 ± 1.6	14.9 ± 4.5^*^, ^#^
TG content, mg/g	24 ± 5	117 ± 49^*^	53 ± 39^#^
**Muscle parameters**			
Gastrocnemius, mg	281 ± 31	299 ± 20	334 ± 18^*^
Soleus, mg	18.7 ± 1.0	16.7 ± 2.0	19.0 ± 2.0
Gastrocnemius TG content, mg/g	12.7 ± 7.2	26.7 ± 16.8	28.3 ± 13.1
**Blood and plasma parameters**			
Glucose, mmol/L	8.30 ± 1.63	9.72 ± 0.64	7.90 ± 1.47
Lactate, mmol/L	1.67 ± 0.19	1.75 ± 0.55	2.22 ± 0.78
NEFAs, mmol/L	0.54 ± 0.12	0.30 ± 0.09^*^	0.58 ± 0.12^#^
Insulin, mU/L	17.5 ± 5.7	58.2 ± 15.5^*^	41.0 ± 19.4
FGF21, pg/mL	<LOD	159 ± 226	131 ± 188
Osteocrin, pg/mL	72.3 ± 34.3	50.9 ± 17.2	87.5 ± 49.5
Osteonectin, ng/mL	6.98 ± 7.21	9.00 ± 1.88	7.09 ± 4.20

*Note*: Data are means ± SD (*n *= 5 or 6).

Abbreviations: AT, adipose tissue; FGF21, fibroblast growth factor 21; <LOD, below the limit of detection; NEFAs, non‐esterified fatty acids; TG, triacylglycerol.

* Statistically significant versus lean.

^#^ Statistically significant versus obese (one‐way ANOVA).

### 2.4 Plasma and tissue collection

Mice were killed by cervical dislocation under isoflurane anaesthesia. Truncal blood was collected into EDTA‐containing tubes for plasma isolation. Liver, gastrocnemius and soleus muscles and AT samples from the epididymal and subcutaneous fat depots were dissected and weighed. Tissue aliquots were used immediately (AT explants) or snap‐frozen in liquid N_2_ and stored at −80°C for subsequent analyses.

### 2.5 AT explants and *ex vivo* lipolysis

Aliquots (∼20 mg) of epididymal or subcutaneous AT were washed with PBS and pre‐incubated on a 96‐well plate in 200 µL of Dulbecco's modified Eagle's medium low glucose (1 g/L; Gibco, UK) containing 2% fatty acid‐free serum albumin (MP Biochemicals; Irvine, CA, USA) at 37°C in a 5% CO_2_ atmosphere for 1 h to release the contents of damaged cells. The explants (*n *= 3 per mouse) were then transferred to new wells and incubated for another 1 h using the same medium and conditions as above. Conditioned medium was used for the measurement of non‐esterified fatty acids (NEFA) using the NEFA‐HR(2) assay (FUJIFILM Wako Chemicals Europe GmbH; Neuss, Germany). Explants were washed in PBS and lysed overnight in a buffer containing 0.3 N NaOH and 0.1% SDS at 55°C with vigorous shaking. The protein content was determined using the bicinchoninic acid assay.

### 2.6 Plasma metabolites and hormones

NEFA levels were measured using the kit from Wako Chemicals Europe GmbH. Glucose and lactate levels were measured in tail blood using the glucometers Contour Plus (Bayer Healthcare AG; Leverkusen, Germany) and Lactate Plus Meter (Nova Biomedical; Waltham, MA, USA), respectively. Plasma insulin levels were measured using an Ultrasensitive Mouse Insulin ELISA kit (Mercodia; Uppsala, Sweden). Myokines, including fibroblast growth factor 21 (FGF21), osteocrin/musclin and osteonectin, were quantified using xMAP technology and the MILLIPLEX Mouse Myokine Magnetic Bead Panel MMYOMAG‐74K (Merck‐Millipore; Burlington, MA, USA). The homeostatic model assessment of insulin resistance (HOMA‐IR) was applied to assess the level of insulin resistance according to the formula: fasting plasma insulin (in mU/L) × fasting plasma glucose (in mmol/L)/22.5.

### 2.7 Triacylglycerol content in the liver and muscle

Tissue TG content (∼50 mg aliquots) was measured in tissue homogenates as before (Pavlisova et al., 2016).

### 2.8 Gene expression analysis

Gene expression was analysed using quantitative real‐time PCR as before (Flachs et al., 2011; Rossmeisl et al., 2020). Transcript levels were normalized using the housekeeping gene eukaryotic translation elongation factor 2 (*Eef‐2*). Primer sequences are listed in the Appendix (Table A1).

### 2.9 Tissue extraction and protein detection

Protein levels in tissue lysates were assessed by western blot (Horakova et al., 2012) using primary antibodies (Cell Signaling Technology, Danvers, MA, USA) against HSL (1:1000; #4107), phospho‐HSL (Ser563 and Ser660; 1:1000; antibodies #4139 and #45804, respectively) and GAPDH (1:2000; #2118; used as the loading control).

### 2.10 Lipidomic analysis

Lipidomic profiling was performed using an untargeted workflow for the lipidome, metabolome and exposome analysis, as before (Janovska et al., 2020; Sistilli et al., 2021); see the Appendix and Table A2 for a list of annotated lipid species.

### 2.11 FAHFA analysis

Samples of plasma (120 µL) and AT (40–50 mg) were extracted according to previously described methods, with several modifications to improve extraction yield and FAHFA detection during LC‐MS analysis (Riecan et al., 2024; Yore et al., 2014); see Tables A3 and A4 for a list of annotated FAHFA in plasma and epididymal AT samples, respectively; see the Appendix for details on methodology. The analysis was conducted blind to the experimental conditions.

### 2.12 Data processing and statistics

Results are means ± SD. Differences were considered significant when *P *≤ 0.05. Normality was assessed using the Shapiro–Wilk test in conjunction with inspection of Q–Q plots. Data that deviated from normality were log_10_‐transformed to meet the assumptions required for parametric analyses better. A one‐way ANOVA followed by Tukey's *post hoc* test was used to compare lean, obese and aged mice at baseline and their running times to exhaustion (GraphPad Prism 10.2 software; La Jolla, CA, USA). A two‐way ANOVA followed by Tukey's or Fisher's least significant difference *post hoc* test was used to compare running mice with their non‐exercising controls and to compare changes over time with baseline values. Student's unpaired *t*‐test was used to compare plasma lactate levels and HSL phosphorylation in running mice vs. non‐exercising controls. The Pearson correlation coefficient was used to assess the strength of the relationships between the selected variables. Lipidomic data analysis was performed using MetaboAnalyst v.6.0 (Pang et al., 2022).

## 3 RESULTS

### 3.1 FAHFA levels in AT and plasma

Initially, we compared lean, obese and aged mice at baseline (Table 1). Both obese and aged mice had higher body weight and adiposity compared with lean animals. Aged mice also had greater gastrocnemius muscle mass than lean mice. However, aged mice had lower epididymal and subcutaneous AT weight, total adiposity and liver TG content, but higher liver glycogen content compared with their obese counterparts. Baseline NEFA levels were reduced in obese mice compared with the other groups. Plasma insulin levels (Table 1) and HOMA‐IR (Figure A2a) were increased by >3‐fold in obese compared with lean mice, and there was a positive correlation between HOMA‐IR levels and body weight (Figure A2b). Baseline plasma myokine levels did not differ between groups (Table 1).

Furthermore, LC‐MS analysis annotated 54 FAHFA regioisomers in epididymal AT and 32 in plasma (Figure A3). Although total FAHFA levels in epididymal AT of obese and aged mice were significantly lower compared with lean animals, plasma levels were comparable between groups (Figure 1a). However, according to the principal component analyses based on all FAHFA species detected in the epididymal AT (Figure 1b) and plasma (Figure 1c), obese mice were clearly separated from the other two groups primarily in plasma (Figure 1c). The reduction in total FAHFA levels observed in AT of obese and aged mice was related to FAHFA containing monounsaturated, but mainly polyunsaturated fatty acids (Figure 1d). For FAHFA containing saturated fatty acids, the majority of PAHPA and PAHSA regioisomers were reduced in aged but not obese mice, whereas all three SAHSA regioisomers were increased in both groups (Figure 1d). Obesity, but not age, was associated with significant but opposing effects on plasma FAHFA regioisomers, with FAHFA containing zero or one double bond mostly being elevated and FAHFA containing more unsaturated fatty acids mostly being decreased (Figure 1e). Regarding the relationship between AT levels of FAHFA regioisomers and the degree of insulin resistance, the strongest negative correlation was found for polyunsaturated FAHFA including 12‐LAHOA, 12‐OAHOA and 13/12‐LAHPA (Figure A2c); in plasma, both negative (e.g., 13‐POHLA) and positive (e.g., 10‐PAHSA) correlations were found (Figure A2d). These results demonstrate the heterogeneity of FAHFA profiles in AT and plasma of lean, obese and aged mice.

**FIGURE 1:**
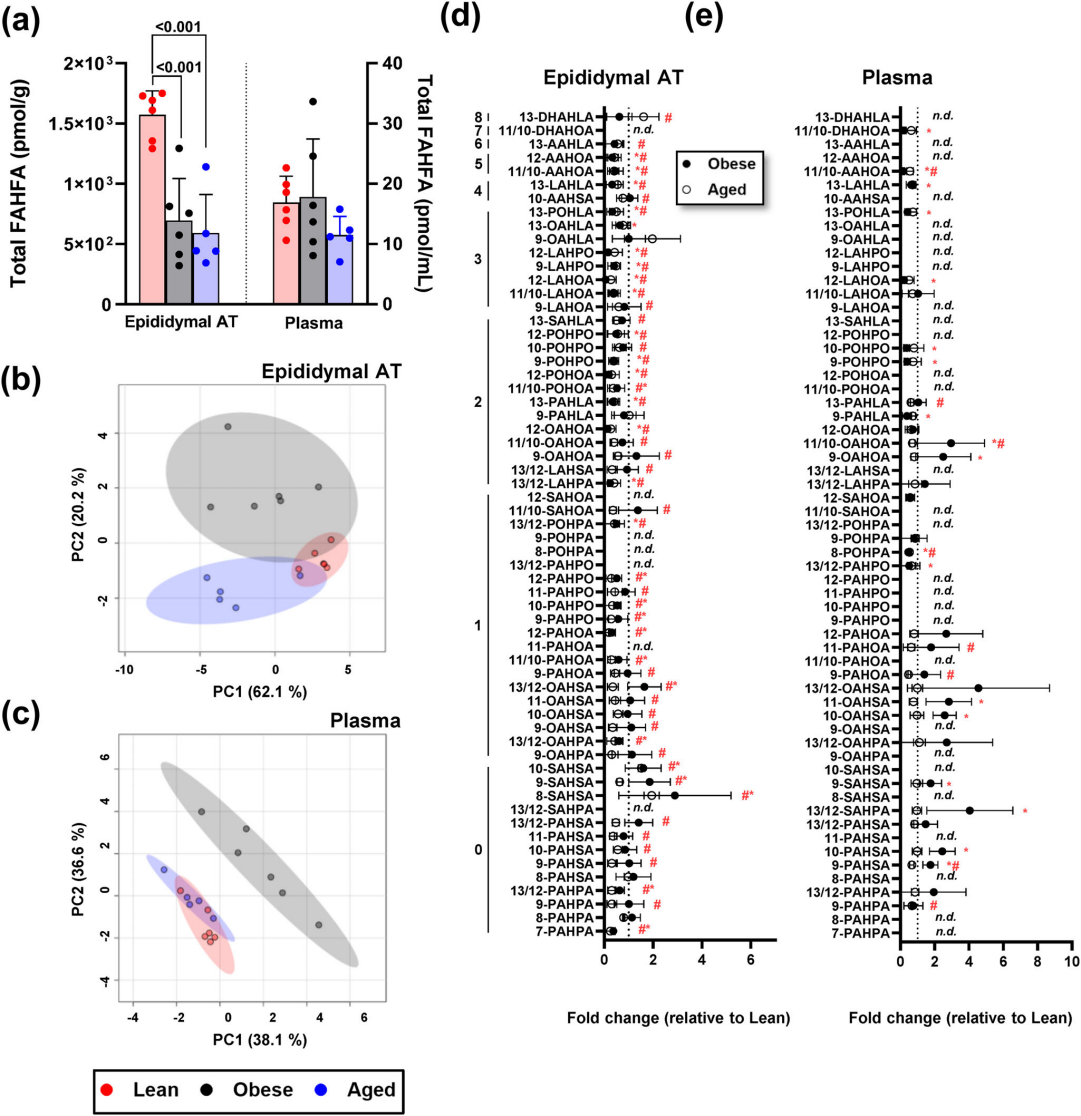
FAHFA levels in epididymal AT and plasma of lean, obese and aged mice measured at baseline, before the onset of acute exercise. (a) Total FAHFA levels in epididymal AT and plasma. Significant *P*‐values are shown in the graph (one‐way ANOVA). (b, c) principal component analysis score plots of identified FAHFAs in epididymal AT and plasma. (d, e) Relative levels (fold change vs. lean) of individual FAHFA regioisomers in obese (filled circles) and aged (open circles) mice in epididymal AT and plasma. *Significant difference obese versus lean; ^#^Significant difference aged versus lean; *P *≤ 0.05 (Student's unpaired *t*‐test; Metaboanalyst 6.0). Results are means ± SD (*n *= 5 or 6). Abbreviations: AT, adipose tissue; FAHFA, fatty acid esters of hydroxy fatty acids; n.d., not detected; PC, principal component.

### 3.2 Changes in FAHFA metabolism‐related gene expression and lipidome in AT

The expression of genes related to FAHFA metabolism was analysed in epididymal AT (Figure 2a,b); compared with lean mice, mRNA levels of *de novo* lipogenesis (DNL)‐related genes associated with FAHFA synthesis were reduced in obese animals, with age having a lesser effect (Figure 2a). The same pattern was observed for lipolysis‐related gene expression (Figure 2b). The expression of genes encoding FAHFA‐degrading enzymes was upregulated in aged mice (Figure 2b).

**FIGURE 2:**
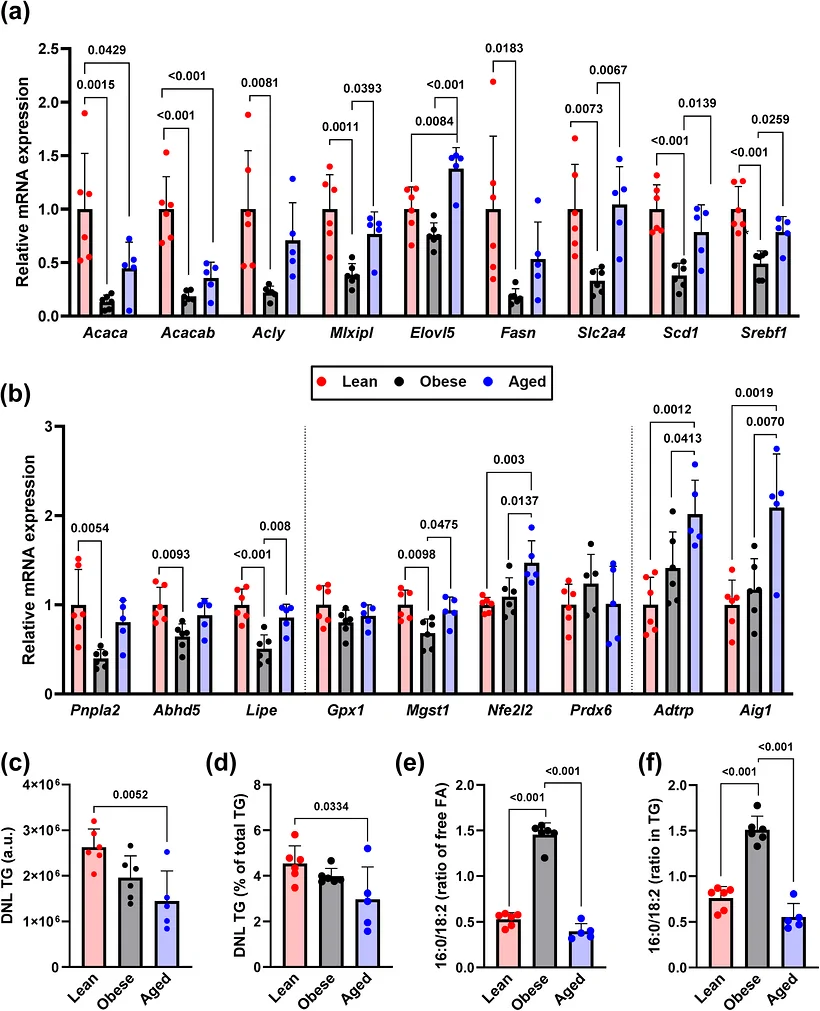
Parameters related to FAHFA metabolism in epididymal AT baseline. (a, b) Gene expression analysis (qPCR) focused on the DNL pathway, in addition to lipolysis, oxidative stress and FAHFA degradation. (c, d) DNL indices based on metabolomic liquid chromatography–mass spectrometry analysis of specific short/medium‐chain TG molecules that represent markers of DNL pathway activity expressed as the absolute and relative content of DNL‐related TG species. (e, f) Alternative markers of DNL pathway activity expressed as the ratio of palmitic acid to linoleic acid in the free fatty acid and TG fractions. Significant *P*‐values are shown on the graph (one‐way ANOVA). Results are means ± SD (*n *= 5–6). Abbreviations: AT, adipose tissue; DNL, *de novo* lipogenesis; FA, fatty acids; FAHFA, fatty acid esters of hydroxy fatty acids; TG, triacylglycerol.

The above data were complemented by AT lipidome analysis (Figure A4; Table A2), focusing on pathways important for FAHFA synthesis and/or degradation. Obesity was associated with decreased AT concentrations of most lipid species (based on a change of ≥2‐fold, *P *< 0.05; Figure A4a), whereas in aged mice significantly fewer lipid metabolites were altered (obese, 188 vs. aged, 75), and most of them were reduced (Figure A4b). Among the lipids whose levels were increased in AT of obese mice were various ceramides, diacylglycerols (DG), phosphatidylglycerols (PG) or ether‐linked phosphatidylcholines (PC; Figure A4a); in aged mice, DG, fatty acids and TG were among the most affected lipid classes (Figure A4b). In obese mice, a set of predominantly downregulated TG, fatty acids and various phospholipid species was observed (vs. lean mice; Figure A4c), whereas in aged animals (Figure A4d) most species originated primarily from the TG, DG and fatty acid classes. Because insulin resistance in AT is linked to reduced DNL and FAHFA synthesis (Moraes‐Vieira et al., 2016; Yore et al., 2014), we placed particular focus on those TG species potentially associated with DNL (Brezinova et al., 2020; Horakova et al., 2023; Sanders et al., 2018). Specifically, we analysed the sum of all TG species containing 38–48 total carbon atoms and no more than three double bonds. These TG species were evaluated both as absolute levels obtained from lipidomic analysis and as their relative proportion within the pool of all detected TG species. These TG species were lower in aged compared with lean mice, both in absolute values (Figure 2c) and relative to all identified TG species (Figure 2d). Another commonly used indicator of DNL activity is the ratio of 16:0 to 18:2 fatty acids (Chong et al., 2008; Hudgins et al., 1996). The 16:0 to 18:2 ratio was calculated from lipidomic analyses based on both detected free fatty acids and their esterified forms in TG molecules. For the latter, the ratio was derived from the abundance of TG species and their corresponding fatty acid composition. This ratio tended to be lower in aged mice than in lean mice, whereas in obese animals it was markedly elevated, in both the non‐esterified (Figure 2e) and esterified (Figure 2f) fractions, probably as a consequence of HFD feeding (Pavlisova et al., 2016; Sistilli et al., 2021).

### 3.3 Acute exercise‐induced lipolysis

Using an optimized protocol for treadmill exercise (Figure 3a), we compared metabolic responses to acute exercise between mouse cohorts. Running time to exhaustion was comparable in lean and aged mice, but lower in obese animals (Figure 3b). All cohorts showed comparable exercise‐induced increases in lactate levels (Figure 3c). Lean mice had elevated plasma NEFA levels at 0 min and 3 h postexercise (Figure 3d). This response was blunted in both obese (Figure 3e) and aged (Figure 3f) animals. Acute exercise stimulated NEFA release from explants of epididymal AT in lean and aged mice, but this effect was most pronounced in lean mice (Figure 3g,i). The stimulatory effect of exercise was completely absent in both epididymal (Figure 3h) and subcutaneous (Figure A5a–c) AT explants from obese mice. Differential effects of exercise were also evident in the level of HSL activation determined in epididymal (Figure 3j,k) and subcutaneous (Figure A5d,e) AT immediately after exercise; however, only lean mice showed a substantial increase in HSL activation, determined based on Ser563 or Ser660 phosphorylation.

**FIGURE 3:**
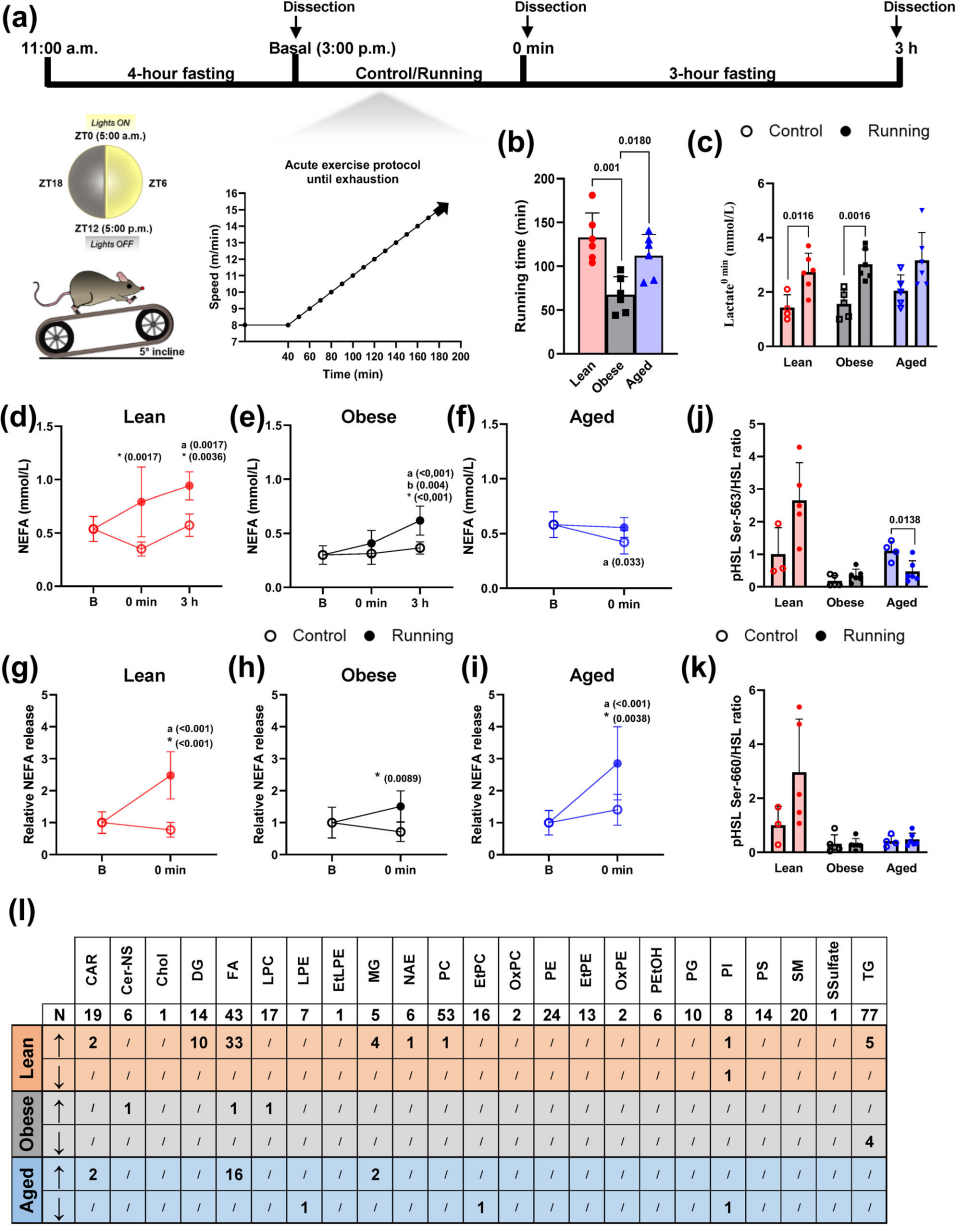
Acute exercise protocol and parameters related to exercise‐induced metabolism and lipolysis. (a) Experimental set‐up. (b) Running time to exhaustion. Significant *P*‐values are shown in the graph (one‐way ANOVA). (c) Blood lactate levels at 0 min. Non‐exercising mice (open circles) were used as controls for running mice (filled circles). Significant *P*‐values are shown in the graph (Student's unpaired *t*‐test). (d–f) Plasma NEFA levels at baseline (‘B’) and at 0 min and 3 h after exercise in lean, obese and aged mice. (g–i) *Ex vivo* analysis of NEFA release from epididymal AT explants from lean, obese and aged mice before exercise (‘B’) or immediately after the end of acute exercise (0 min). Results are means ± SD (*n *= 5 or 6) and are expressed as the fold change from B. *Significant difference versus corresponding non‐exercising controls. ^a^Significant difference versus baseline (‘B’). ^b^Significant difference versus exercising group at 0 min. *P *≤ 0.05 (two‐way ANOVA). (j, k) Western blot analysis of HSL phosphorylation at Ser563 and Ser660 in epididymal AT of mice immediately after exercise (0 min). Results are means ± SD (*n *= 3–5) and are expressed as the fold change from ‘lean, non‐exercising controls’. Significant *P*‐values are shown in the graph (Student's unpaired *t*‐test). (l) Overview of the main lipid classes detected and the number of significantly altered lipid species within each class; *P *≤ 0.05 (Student's unpaired *t*‐test; Metaboanalyst 6.0). Results are means ± SD (*n *= 4–6). Abbreviations: CAR, acylcarnitine; Cer‐NS, ceramide non‐hydroxy fatty acid‐sphingosine; Chol, cholesterol; DG, diacylglycerol; EtherPC, ether‐linked phosphatidylcholine; EtherPE, ether‐linked phosphatidylethanolamine; FA, free fatty acid; HSL, hormone‐sensitive lipase; LPC, lysophophatidylcholine; LPE, lysophosphatidylethanolamine; EtherLPE, ether‐linked lysophosphatidylethanolamine; MG, monoacylglycerol; NAE, *N*‐acyl ethanolamine; OxPC, oxidized phosphatidylcholine; OxPE, oxidized phosphatidylethanolamine; PC, phosphatidylcholine; PE, phosphatidylethanolamine; PEtOH, phosphatidylethanol; PG, phosphatidylglycerol; PI, phosphatidylinositol; PS, phosphatidylserine; SM, sphingomyelin; SSulfate, sterol sulfate; TG, triacylglycerols.

We also measured plasma levels of the hormones FGF21, osteocrin and osteonectin, which are hormones previously shown to be involved in the regulation of fatty acid oxidation or lipolysis (Arner et al., 2008; Choi et al., 2023; Mukherjee et al., 2020). In lean mice, exercise caused a time‐dependent increase in plasma FGF21 levels, which rose to >450 pg/mL at 3 h postexercise (Figure A6a). Obese and aged mice had higher baseline levels of FGF21 (Table 1), which did not increase with exercise (Figure A6a,c). Plasma osteocrin (Figure A6d–f) and osteonectin (Figure A6g–i) levels did not change in response to acute exercise.

Next, an untargeted lipidomic analysis was performed on epididymal AT collected immediately after exercise. Of the 23 identified lipid classes, the main classes whose concentrations increased after exercise were DG, fatty acids and monoacylglycerols (MG; Figure 3l), indicative of active lipolysis (Arner et al., 1976). Within these lipid classes, the highest number of species showing a significant increase in epididymal AT following exercise was observed in lean mice (Figure 3l).

### 3.4 The effect of acute exercise on plasma FAHFA levels

In comparison to non‐exercising controls, total FAHFA levels in lean mice remained unchanged after exercise (Figure 4a), similar to obese and aged mice (Figure 4b,c). However, exercise affected plasma levels of individual FAHFA regioisomers in all mouse cohorts (Figure 4d–f). Thus, in lean mice (Figure 4d), a total of 11 FAHFA regioisomers changed their plasma concentrations after exercise (either increased or decreased), whereas in obese (Figure 4e) and aged (Figure 4f) mice, eight regioisomers were elevated and four regioisomers decreased. Interestingly, 13/12‐OAHSA was increased immediately after exercise in both lean and obese mice but not in aged mice, whereas 11/10‐DHAHOA and 11/10‐AAHOA were inversely regulated by exercise in lean and obese mice. Plasma levels of 9‐ and 10‐PAHSA, which are lipokines with documented insulin‐sensitizing properties, remained either unchanged or decreased in response to acute exercise.

**FIGURE 4:**
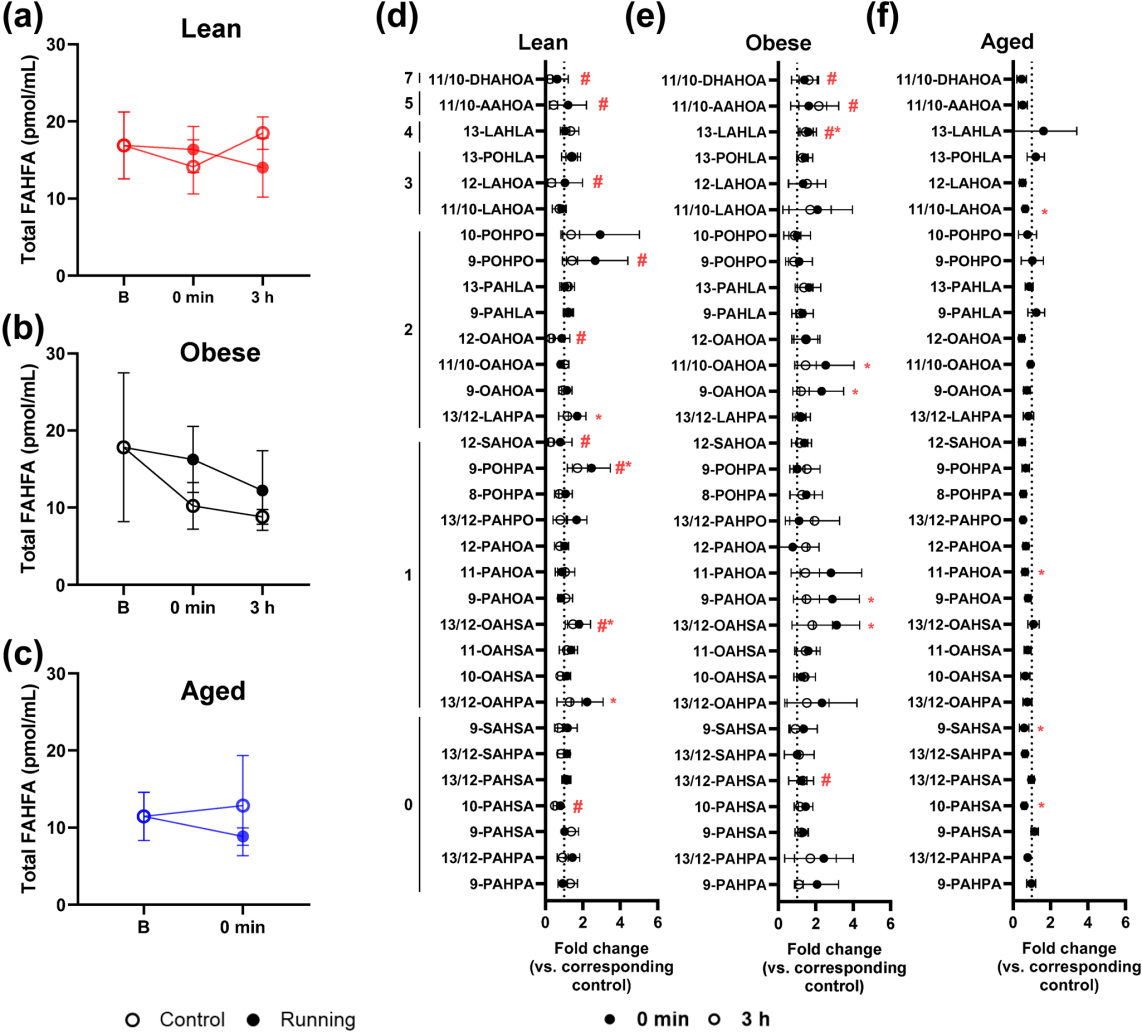
Changes in plasma FAHFA levels in response to exercise. (a–c) Total FAHFA levels in plasma measured at baseline (‘B’) and at 0 min and 3 h after exercise in lean, obese and aged mice. (d–f) Plasma levels of individual FAHFA regioisomers in lean, obese and aged mice at 0 min (filled circles) and 3 h (open circles) postexercise, expressed as a fold change to the mean levels of the respective FAHFA found in the corresponding non‐exercising controls. *Significant difference versus corresponding non‐exercising controls at 0 min; ^#^Significant difference versus corresponding non‐exercising controls at 3 h; *P *≤ 0.05 (Student's unpaired *t*‐test; Metaboanalyst 6.0). Results are means ± SD (*n *= 4–6). Abbreviation: FAHFA, fatty acid esters of hydroxy fatty acids.

### 3.5 Exercise‐induced changes in FAHFA levels in AT

Initially, we analysed total free FAHFA levels in epididymal AT in running and non‐exercising animals. In lean mice, AT FAHFA levels increased immediately after exercise and were elevated further at 3 h postexercise (Figure 5a). At 3 h time point, FAHFA levels also increased to a lesser extent in the non‐exercising controls (Figure 5a). Total FAHFA levels did not increase in AT of obese and aged mice after exercise (Figure 5b,c). The number of individual FAHFA regioisomers whose levels were significantly altered by exercise was by far the highest in lean mice compared with other groups (Figure 5d–f). In lean mice, of the 54 FAHFA regioisomers detected in epididymal AT, 19 were elevated immediately after exercise, whereas 18 were elevated at 3 h postexercise, when compared with non‐exercising controls (Figure 5d). The FAHFA regioisomers whose levels changed the most and consistently in response to exercise in lean mice included 13/12‐regioisomers from different FAHFA subfamilies, all of which showed a marked increase immediately after exercise; moreover, none of the detected regioisomers was reduced in response to exercise (Figure 5d). This contrasts with obese and aged mice, in which the levels of FAHFA regioisomers that responded to exercise were generally reduced, and the number of regioisomers affected by exercise was much lower in obese and especially aged mice (Figure 5e,f). In summary, obesity and ageing negatively affected the ability of acute exercise to increase free FAHFA levels in AT.

**FIGURE 5:**
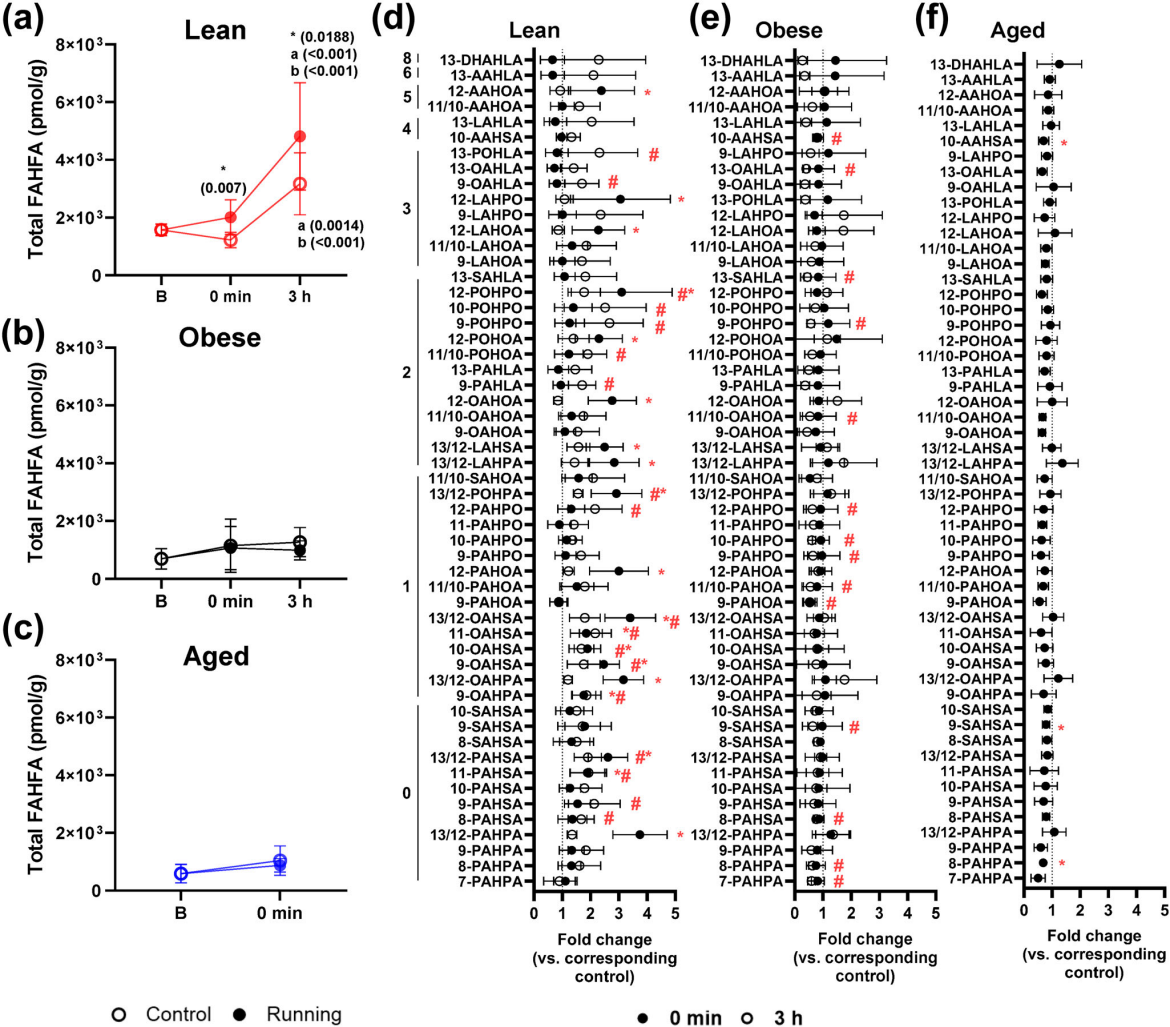
Exercise‐induced changes in FAHFA levels in the epididymal AT. (a–c) Total FAHFA levels in epididymal AT measured at baseline (‘B’) and at 0 min and 3 h after exercise in lean, obese and aged mice. *Significant difference versus corresponding non‐exercising controls; ^a^significant difference versus baseline (‘B’); ^b^significant difference versus exercising group at 0 min; *P *≤ 0.05 (two‐way ANOVA). (d–f) Epididymal AT levels of individual FAHFA regioisomers in lean, obese and aged mice at 0 min (filled circles) and 3 h (open circles) postexercise, expressed as a fold change to the mean levels of the respective FAHFA found in the corresponding non‐exercising controls. *Significant difference versus corresponding non‐exercising controls at 0 min; ^#^Significant difference versus corresponding non‐exercising controls at 3 h; *P *≤ 0.05 (Student's unpaired *t*‐test; Metaboanalyst 6.0). Results are means ± SD (*n *= 4–6). Abbreviations: AT, adipose tissue; FAHFA, fatty acid esters of hydroxy fatty acids.

### 3.6 The relative effects of acute exercise versus fasting alone on the levels of FAHFA and lipolytic intermediates in AT of lean mice

Next, we focused on elucidating the relative effects of exercise and fasting alone on FAHFA and lipolytic intermediate levels in AT of lean mice (Figure 6a–f). Although fasting represents the main lipolytic stimulus in non‐exercising mice, the level of lipolysis might be affected by both exercise itself and a parallel fasting state in running mice. In non‐exercising control animals, immediately after exercise, total FAHFA levels decreased slightly but significantly (Student's unpaired *t*‐test), whereas levels of lipolytic intermediates (i.e., free fatty acids, DG and MG) were unchanged compared with baseline (Figure 6a). In running mice, the levels of all lipolytic intermediates increased, but FAHFA levels remained unchanged immediately after exercise (Figure 6b). At 3 h postexercise, in control mice, both FAHFA and lipolytic intermediates levels were elevated ∼2‐fold compared with baseline (Figure 6c); the FAHFA levels even tended to be higher (∼1.5‐fold increase; *P *= 0.058) compared with those measured in running mice immediately after exercise (Figure 6d), despite no differences in lipolytic intermediate levels. However, running mice at 3 h postexercise showed further potentiation of lipolysis, with levels of lipolytic intermediates elevated ≤6‐fold and FAHFA levels ≤3‐fold above baseline (Figure 6e). This delayed stimulatory effect of exercise was clearly evident when compared with the state immediately after exercise (Figure 6f); however, the relative increase in lipolytic intermediates was noticeable only at the MG level (∼2.5‐fold increase; Figure 6f).

**FIGURE 6:**
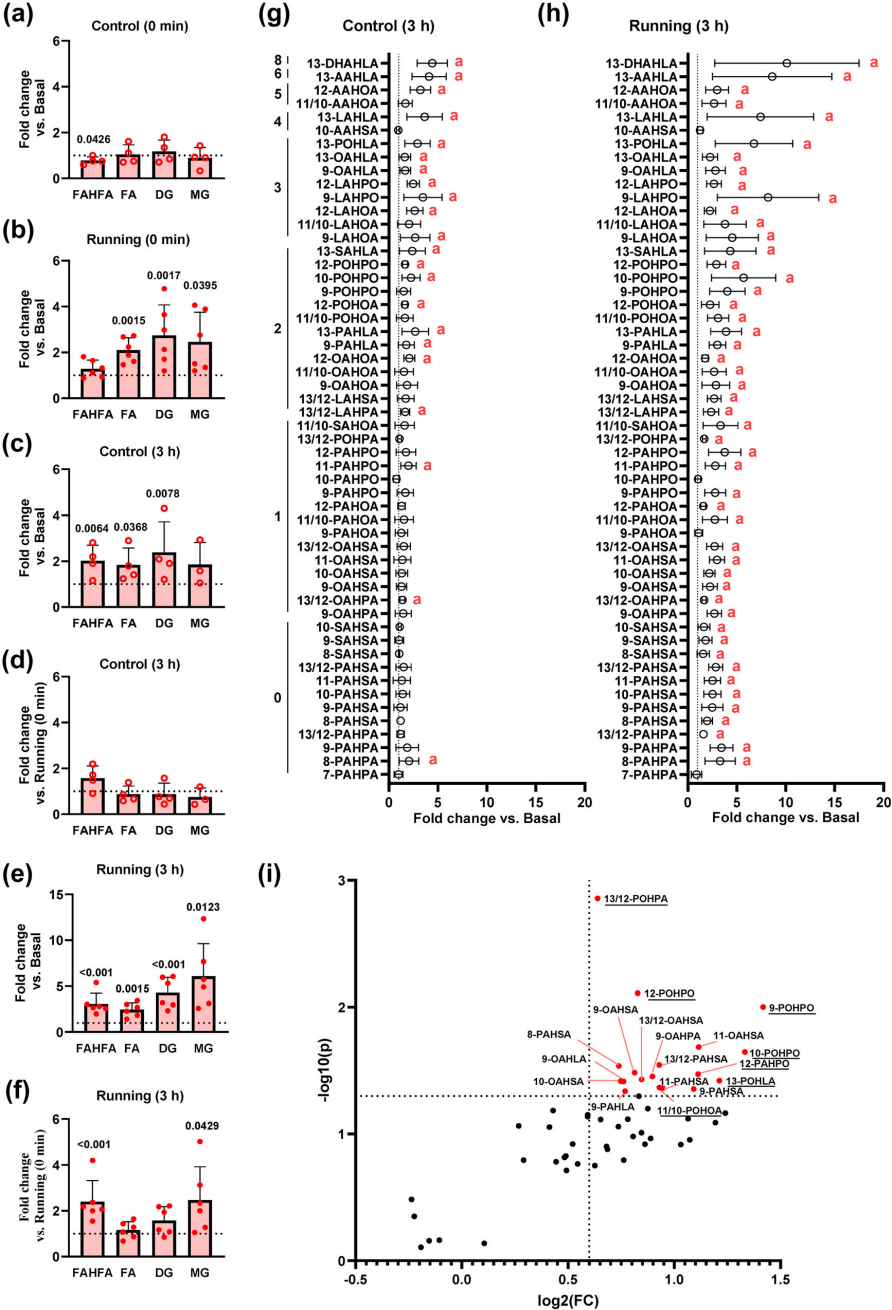
Relative effects of exercise and fasting alone on the levels of FAHFA and lipolytic intermediates in epididymal AT of lean mice. (a–f) Relative levels of total (free) FAHFA, (free) fatty acids, DG and MG in epididymal AT of lean mice exposed or not to acute exercise. Significant *P*‐values (Student's unpaired *t*‐test) are shown on the graph relative to the corresponding groups indicated on the *y*‐axis (marked by a dotted line). (g and h) Levels of individual FAHFA regioisomers in epididymal AT of lean mice measured at the 3 h time point in groups of non‐exercising controls and running mice and expressed as a fold change from baseline. ^a^Significant difference versus baseline; *P* ≤ 0.05 (Student's unpaired *t*‐test; Metaboanalyst 6.0). Results are means ± SD (*n *= 4–6). (i) Volcano plot showing FAHFA regioisomers that are specific to the effect of exercise (vs. fasting alone) at the 3 h time point; the magnitude of change, shown as log_2_(fold change) on the *x*‐axis, is plotted against the statistical significance of that change [−log_10_(*P*‐value)] shown on the *y*‐axis. Abbreviations: AT, adipose tissue; DG, diacylglycerols; FA, (free) fatty acids; FAHFA, fatty acid esters of hydroxy fatty acids; MG, monoacylglycerols.

Regarding the relative effects of exercise and fasting alone on individual FAHFA regioisomers in AT of lean mice at 3 h postexercise (vs. baseline; Figure 6g,h), fasting in control mice had a lesser effect than exercise, because it stimulated lower numbers of FAHFA and mainly those containing at least two double bonds (Figure 6g). In contrast, 50 of the 54 detected regioisomers across all FAHFA families increased as a result of exercise, with the fold change being much greater in comparison to those stimulated by fasting alone (Figure 6h). Some hydroxylinoleic acid (HLA)‐containing FAHFA, such as 13‐POHLA, 13‐LAHLA, 13‐AAHLA and 13‐DHAHLA, were strongly elevated in both groups, whereby, for example, 13‐DHAHLA showed a 5‐ and 10‐fold increase in control and running mice, respectively (Figure 6h). The FAHFA regioisomers that were significantly elevated in AT of non‐exercising controls at the 3 h time point (Figure 6g) did not correspond to the categories of (free) fatty acids that were elevated in AT, because the latter included primarily saturated and monounsaturated fatty acids (Figure A7). In contrast, the vast majority of fatty acids in AT of exercising mice were elevated at this time point (Figure A7).

The exercise‐specific effect on FAHFA regioisomer levels in epididymal AT 3 h postexercise was determined using a volcano plot (Figure 6i); thus, 18 FAHFA regioisomers showed significantly greater increases attributable to exercise. These FAHFA primarily included those from the POHPO family (e.g. 9‐POHPO, 10‐POHPO and 12‐POHPO) that were among the highest ranked regioisomers based on fold change and/or *P*‐value (Figure 6i). Other FAHFA families, of which always three or four regioisomers were more affected by exercise, included OAHSA and PAHSA (Figure 6i). Thus, in terms of stimulating free FAHFA levels in AT, acute exercise elicits a broader and stronger response compared with the effects of fasting alone.

## 4 DISCUSSION

In this study, we analysed the effect of a single bout of exercise on the metabolome and FAHFA lipidome in previously untrained mice, focusing on the regulation of FAHFA metabolism, metabolic response to acute exercise, and the impact of obesity and ageing. The study benefitted from the use of non‐exercised control groups, assessment of both plasma and AT samples, targeted FAHFA regioisomer analysis, and *ex vivo* AT lipolysis measurements. A key finding was that both obesity and ageing impaired the AT response to acute exercise, as evidenced by reduced activation of lipolysis and diminished increases in tissue FAHFA levels.

Obesity and ageing led to a significant reduction in total AT FAHFA levels, but regulated individual FAHFA in a depot‐ and regioisomer‐specific manner. This aligns with prior studies reporting a decrease in PAHSA regioisomers in obese mice subcutaneous AT but little or no change (or even an increase) in gonadal AT (Yore et al., 2014). Ageing selectively modulates FAHFA in visceral AT, with distinct profiles among regioisomers throughout the lifespan (Zhu et al., 2020). In contrast to those results, we found that nearly all FAHFA regioisomers decreased in epididymal AT of aged (18‐month‐old) mice, although SAHSA family members increased with age in both studies. Our aged mice correspond to an early stage of ageing in murine models. This age was selected because it was expected to provide sufficient ageing‐associated metabolic impairment while still preserving exercise capacity comparable to that of young mice (Pajski et al., 2025). Moreover, previous studies have reported that both lean mass and total body mass increase with age in C57BL/6 mice (Reynaga et al., 2025), which is consistent with our observation that aged mice exhibited greater gastrocnemius muscle mass and total body mass compared with lean mice. It is possible that mice in a more advanced stage of ageing will exhibit even more pronounced differences compared with lean mice, particularly with regard to baseline FAHFA levels, exercise capacity and the metabolic effects of exercise.

We analysed gene expression relevant to FAHFA metabolism in AT: DNL, oxidative stress, transacylation, lipolysis and degradation (Brejchova et al., 2021; Erikci Ertunc et al., 2020; Kuda et al., 2018; Patel et al., 2022; Yore et al., 2014). Reductions in DNL and lipolysis‐related gene expression, especially among obese mice, matched their lower total free FAHFA, indicating that obesity combined with HFD impairs DNL and lipolysis more than physiological ageing. Indices for DNL activity showed decreased activity even in aged mice. In addition, aged mice had increased expression of FAHFA hydrolases androgen‐dependent tissue factor pathway inhibitor regulating protein (*Adtrp*) and androgen‐induced gene 1 (*Aig1*) (Erikci Ertunc et al., 2020; Parsons et al., 2016), suggesting higher FAHFA degradation as a contributor to the observed decrease in FAHFA levels. Chronic inflammation in AT has been associated with increased AIG1 activity, resulting in lower FAHFA and insulin resistance (Yan et al., 2024). This mechanism might also be relevant in our aged cohort. However, changes in gene expression do not fully explain the variability seen among individual FAHFA regioisomers; for example, oxidative stress in obese (Furukawa et al., 2004) and possibly aged mice could selectively stimulate specific FAHFA synthesis in baseline conditions.

Substantial heterogeneity was seen in plasma: 8 of 32 FAHFA regioisomers were elevated in obese mice, but none in aged counterparts. We found that 9‐ and 10‐PAHSA, along with 13/12‐SAHPA and 9‐SAHSA, were among the four saturated FAHFA increased in the plasma of obese mice, as previously reported (Yore et al., 2014). Notably, increased levels of 9‐SAHSA have been found in the breast milk of obese mothers (Dong et al., 2025). The different interplay between FAHFA synthesis and breakdown contributes to the unique FAHFA profiles observed in AT and plasma across mouse cohorts. Our exercise protocol was optimized to maximize lipolysis, hypothesising that exercise‐driven NEFA increases would correspond to changes in tissue and plasma FAHFA levels. Low‐intensity exercise relies more on lipids, especially AT‐derived NEFA for muscle energy (Brooks, 1997; Brooks & Mercier, 1994). Previous research showed that AT lipolysis does not increase further at moderate or high exercise intensity compared with low intensity (Romijn et al., 1993). Our treadmill protocol combined with mild fasting resulted in a 50%–80% rise in plasma lactate, consistent with prior data on low‐intensity exercise (Brandt et al., 2017; Fuller et al., 2019). Lactate might indicate exercise intensity alongside maximal O_2_ uptake (Lonbro et al., 2019), and similar lactate responses immediately postexercise in lean, obese and aged mice suggest comparable relative exercise intensity.

Consistent with our hypothesis, lean mice showed increases in both NEFA and AT FAHFA following exercise. However, only AT FAHFA increased; plasma FAHFA remained unchanged. This indicates that FAHFA release from adipocytes might differ from NEFA transport, potentially at the level of the CD36 receptor; CD36 is linked to biosynthesis of polyunsaturated FAHFA (Kuda et al., 2018), and the interaction of 5‐POHSA with CD36 can promote calcium signalling (Muthuswamy et al., 2024). In lean mice, non‐exercising control animals studied at 3 h postexercise had higher AT FAHFA compared with mice analysed immediately after exercise, despite similar lipolysis. This can be attributed to an increased activity of HSL, a known FAHFA hydrolase (Brejchova et al., 2021), after exercise, which might promote lipolysis and simultaneous FAHFA breakdown. The hydrolases ADTRP and AIG1 might also play a role (Erikci Ertunc et al., 2020; Riecan et al., 2024). The possibility of greater FAHFA synthesis during or after exercise, perhaps linked to acute inflammation, is supported by evidence that inflammation induces hydroxy fatty acids that convert to anti‐inflammatory FAHFA via ATGL (Ertunc et al., 2024). In addition, fasting itself can promote ATGL‐driven FAHFA synthesis (Santoro et al., 2026). Therefore, the observed differences between mice analysed immediately after exercise (0 h) and those analysed 3 h later are likely to reflect a combination of acute and delayed exercise‐induced effects superimposed on a fasting background.

We observed uneven responses among FAHFA regioisomers to exercise in AT and plasma, especially in lean mice. Notably, all detected 13/12‐FAHFA regioisomers rose sharply immediately after exercise in AT, suggesting enzyme affinity for these regioisomers, probably via ATGL‐mediated FAHFA release from TG estolides (Paluchova et al., 2020). Three such regioisomers (13/12‐OAHPA, 13/12‐OAHSA and 13/12‐LAHPA) also increased in plasma. Acute rises in FAHFA might regulate fatty acid esterification or acylglycerol remodelling or might fine‐tune lipolysis (Paluchova et al., 2020). The role of AT in systemic exercise adaptation warrants further exploration. In contrast, the FAHFA response to acute exercise was strongly diminished in AT from obese and aged mice, probably owing to impaired lipolysis. Obesity is associated with reduced β‐adrenergic receptor expression and impaired stimulation of adenylyl cyclase (Collins et al., 1997), alongside upregulated α‐adrenergic receptors in AT, constituting an adaptive response to prevent lipotoxicity (Laurens et al., 2020; Rossmeislova et al., 2024; Stich et al., 2000). Additional factors limiting lipolysis in obesity might include lower expression of atrial natriuretic peptide receptor A, increased clearance receptor C and diminished tissue blood flow (Crandall et al., 1984; Kovacova et al., 2016; Laurens et al., 2020; Thompson et al., 2012; West et al., 1987). Our data showed a reduced response of plasma NEFA to acute exercise in obese and aged mice, aligning with these mechanisms of impaired lipolysis. However, functional AT lipolysis does not appear essential for increasing plasma FAHFA postexercise. Other studies have shown that lipolytic stimuli, such as starvation or cold exposure (Paluchova et al., 2020; Yore et al., 2014), or hydrolase inactivation in AT (Erikci Ertunc et al., 2020), do not necessarily raise plasma FAHFA levels. The relationship between tissue‐specific FAHFA synthesis and circulating FAHFA levels remains unknown, and it is likely that multiple tissues contribute to the circulating FAHFA pool.

This study has several limitations. Our results were obtained only from preclinical models. Given the absence of human studies conducted in the same experimental conditions, it is not possible to draw conclusions regarding the translatability of our data. Another potential limitation is that female mice were not used, because oestrus might affect AT function. Moreover, female mice have been shown to have higher AT levels of FAHFA owing to their inverse correlations with testosterone and *Adtrp* mRNA levels, both of which are lower in females (Riecan et al., 2024). Female mice are also known to exhibit differences in the regulation of lipolysis, in addition to overall differences in lipid metabolism compared with male mice (Benz et al., 2012; Many et al., 2024). Furthermore, a small age mismatch between lean and obese mice (Table 1) represents an additional potential limitation of the study. Nevertheless, both groups consist of adult mice (aged ∼4.5–5 months), hence it is unlikely that this slight difference in their age would have any significant impact on the observed results. In addition, the shorter running time to exhaustion was observed in obese mice compared with the lean group. However, no difference in running time to exhaustion was detected between young lean and aged mice, suggesting that the observed differences in lipolysis and postexercise AT FAHFA levels are more likely to be attributable to metabolic impairments rather than differences in exercise capacity. Lastly, given the complex nature of FAHFA metabolism and the potential involvement of oxidative stress in their biosynthesis (Kuda et al., 2018; Riecan et al., 2022), higher‐intensity exercise could affect FAHFA metabolism differently.

## 5 CONCLUSION

Our data show that acute exercise generally increases AT levels of NEFA and FAHFA in previously untrained lean male mice, whereas this effect is blunted by obesity and ageing, probably owing to impaired lipolysis and/or increased FAHFA degradation. These findings offer new insights into the complex interplay of exercise, obesity and ageing in lipid metabolism and support further exploration of FAHFA regulation for metabolic health.

## AUTHOR CONTRIBUTIONS

Marko Mitrovic contributed to data collection and analysis and interpretation of results and drafted the manuscript. Olga Horakova participated in the study design and contributed to data collection and analysis and interpretation of results. Martin Riecan contributed to data analysis. Veronika Kleinova contributed to data collection and analysis. Tomas Cajka contributed to data collection and analysis. Lenka Rossmeislova participated in the study design and interpretation of results. Ondrej Kuda contributed to data analysis. Martin Rossmeisl obtained funding, participated in the study design, contributed to data collection and interpretation of results and drafted the manuscript. All authors contributed to the editing of the manuscript, approved the final version of the manuscript and agree to be accountable for all aspects of the work in ensuring that questions related to the accuracy or integrity of any part of the work are appropriately investigated and resolved. All persons designated as authors qualify for authorship, and all those who qualify for authorship are listed.

## CONFLICT OF INTEREST

None declared.

## GENERATIVE AI STATEMENT

No generative AI tools were used in the preparation of this manuscript.

## Figures and Tables

**FIGURE A1:**
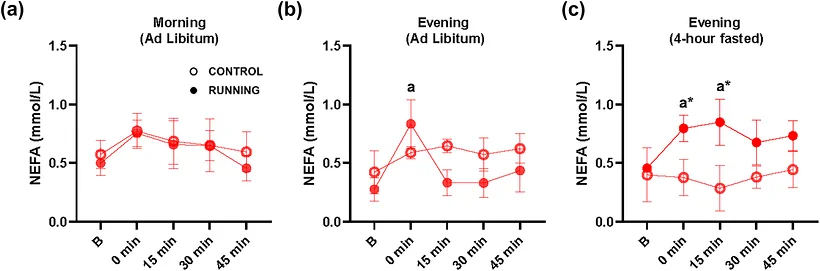
Pilot experiments to determine the optimal conditions that provide the greatest lipolytic stimulus. Plasma NEFA levels were measured in lean mice exposed to various conditions, including the intensity, duration and timing of acute exercise or the availability of food prior to exercise. Mice with free access to food were exposed to one bout of exercise in the morning (9:00 h) or evening (16:00–19:00 h), with the latter timing also tested in animals after a 4 h period without access to food. Non‐exercising mice were used as controls (empty circles) for running mice (filled circles). Data are means ± SD (*n *= 3 or 4). ^a^Significant difference vs. baseline (‘B’); *significant difference versus corresponding non‐exercising controls; *P *≤ 0.05 (two‐way ANOVA). Abbreviations: NEFA, non‐esterified fatty acids.

**FIGURE A2:**
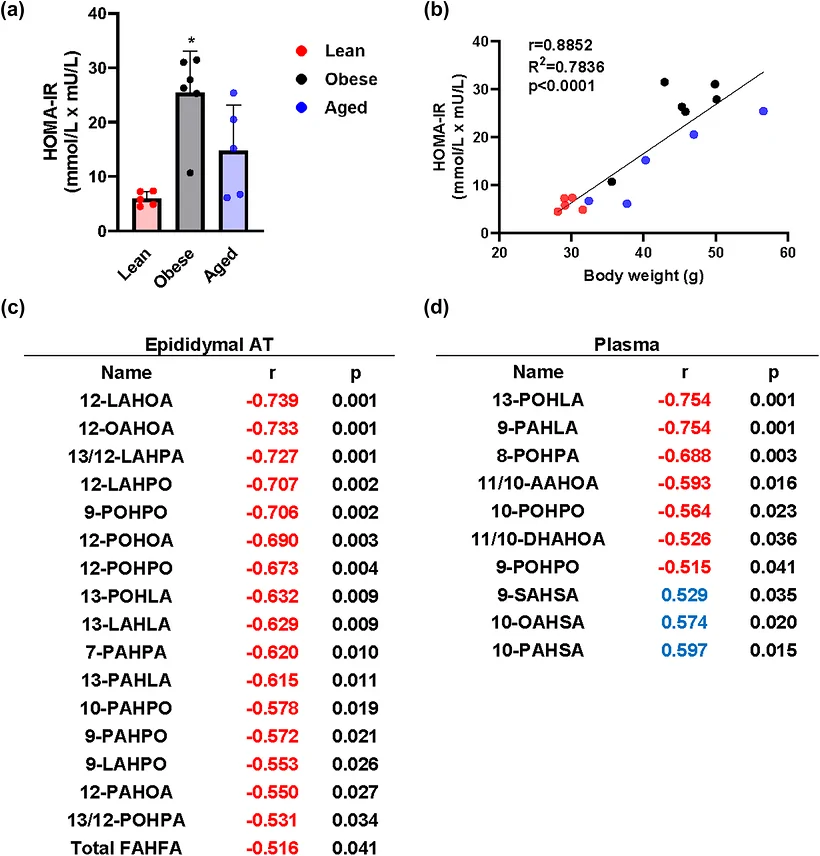
Basal characteristics related to insulin sensitivity and FAHFA profiles in AT and plasma. (a) HOMA‐IR in lean, obese and aged mice. Results are means ± SD (*n *= 5–6). *Significant difference versus lean mice; *P *≤ 0.05 (one‐way ANOVA). (b) The relationship between body weight and HOMA‐IR levels. (c, d) FAHFA regioisomers in epididymal AT and plasma, whose levels show a significant correlation with HOMA‐IR (combined data from all groups). The Pearson correlation coefficient was used to assess the strength of the relationships between the variables. Abbreviations: AT, adipose tissue; FAHFA, fatty acid esters of hydroxy fatty acids; HOMA‐IR, homeostatic model assessment of insulin resistance.

**FIGURE A3:**
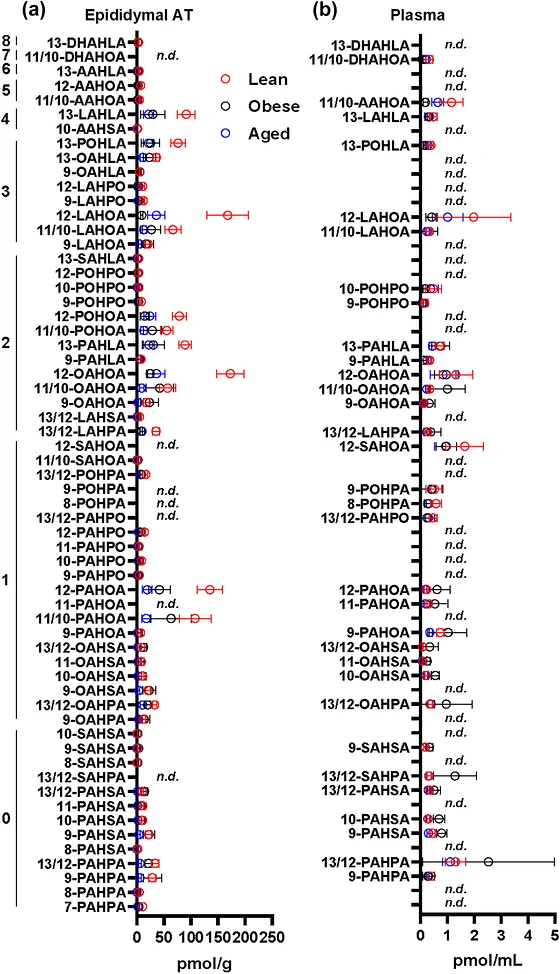
Absolute FAHFA levels at baseline. FAHFA regioisomer levels were determined by liquid chromatography–mass spectrometry analysis in epididymal AT and plasma of lean (red circles), obese (black circles) and aged (blue circles) mice before the onset of acute exercise, that is at 15:00 h, after a 4 h period without access to food. Results are means ± SD (*n *= 5 or 6). Abbreviations: AT, adipose tissue; FAHFA, fatty acid esters of hydroxy fatty acids; n.d., not detected

**FIGURE A4:**
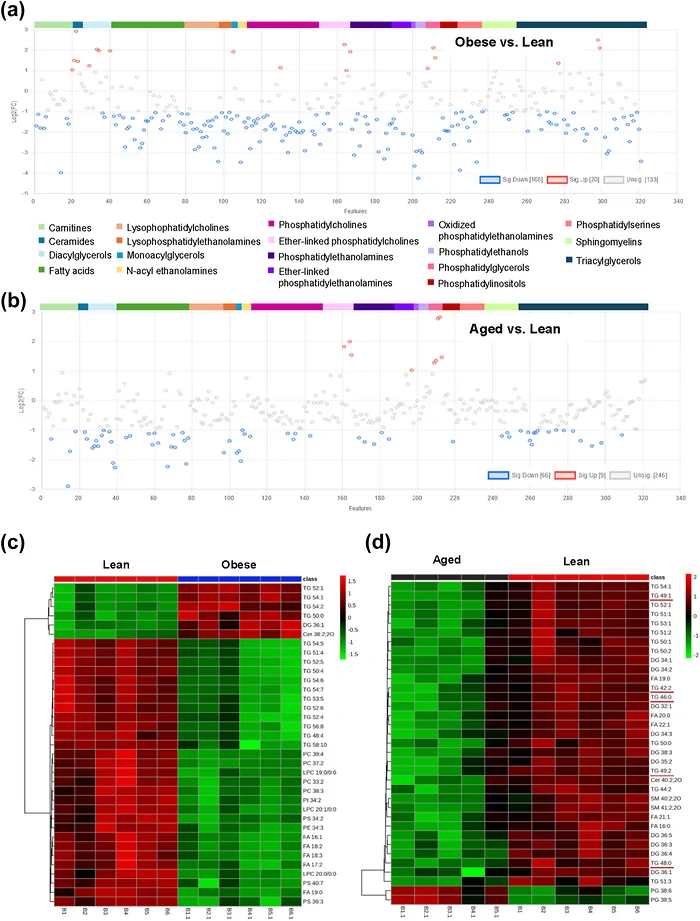
Analysis of epididymal AT lipidome in lean, obese and aged mice at baseline. (a, b) Relative levels of annotated lipid species identified in the epididymal AT of obese and aged mice, expressed as a fold change from levels found in lean counterparts (*n *= 5 or 6). Lipids showing significant changes are shown as blue circles (downregulated) or red circles (upregulated); grey circles indicate non‐significant changes. (c, d) Heat‐maps showing the top 35 most differentiating lipids in lean versus obese mice (c) and lean versus aged mice (d) (Student's unpaired *t*‐test; Metaboanalyst 6.0). Abbreviations: AT, adipose tissue; Cer, ceramides; DG, diacylglycerols; FA, fatty acids; LPC, lysophosphatidylcholines; PC, phosphatidylcholines; PE, phosphatidylethanolamines; PI, phosphatidylinositols; PS, phosphatidylserines; SM, sphingomyelins; TG, triacylglycerols.

**FIGURE A5:**
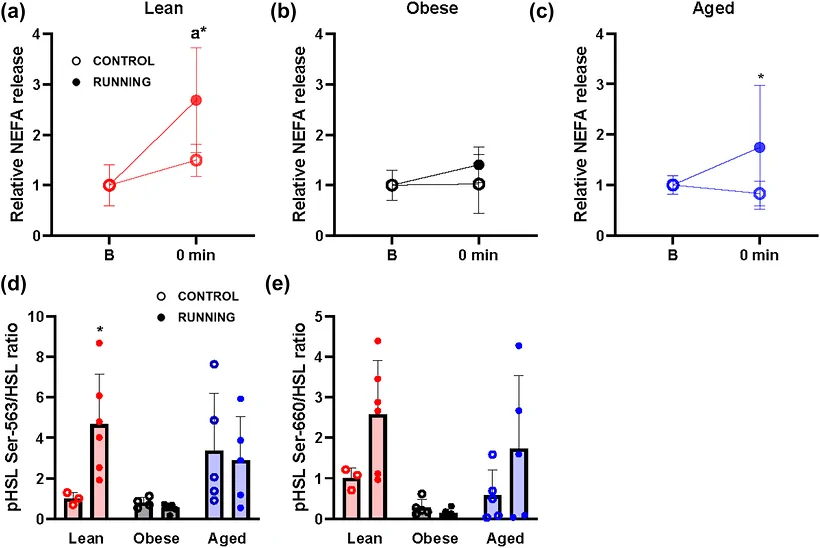
*Ex vivo* analysis of lipolysis in subcutaneous AT explants. (a–c) Lipolysis expressed as the amount of NEFA released into the medium from subcutaneous (inguinal) AT explants from lean, obese and aged mice before exercise (i.e., at baseline; ‘B’) or immediately after the end of acute exercise (0 min). Non‐exercising mice (open circles) were used as controls for running mice (filled circles). Results are means ± SD (*n *= 4–6) and are expressed as a fold change from ‘B’. *Significant difference versus corresponding non‐exercising controls; ^a^significant difference versus baseline (‘B’); *P *≤ 0.05 (two‐way ANOVA). (d, e) Western blot analysis of HSL phosphorylation at Ser563 and Ser660 in inguinal AT of mice immediately after exercise (0 min). Results are means ± SD (*n *= 3–6) and are expressed as a fold change from lean, non‐exercising controls. *Significant difference vs. corresponding non‐exercising controls; *P *≤ 0.05 (Student's unpaired *t*‐test). Non‐exercising mice (empty circles) were used as controls for running mice (filled circles). Abbreviations: AT, adipose tissue; HSL, hormone‐sensitive lipase; NEFA, non‐esterified fatty acids.

**FIGURE A6:**
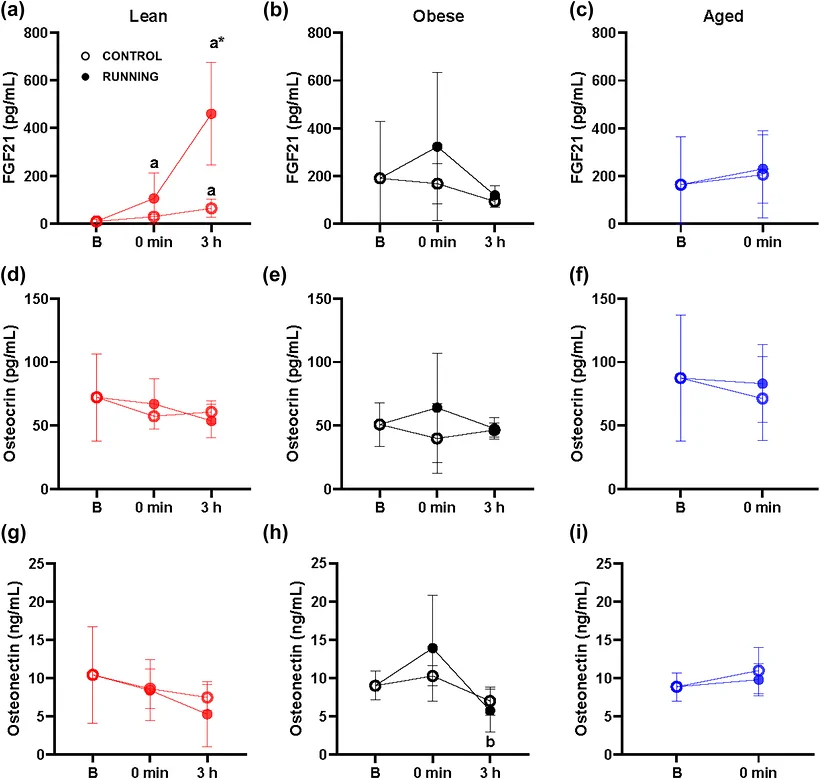
Effect of exercise on plasma myokine levels. (a–c) Plasma levels of FGF21 in lean, obese and aged mice, measured before exercise (baseline; ‘B’), immediately after acute exercise (0 min) and 3 h postexercise (3 h; ‘aged’ mice were not analysed at this time point). (d–f) Plasma levels of osteocrin in lean, obese and aged mice. (g–i) Plasma levels of osteonectin in lean, obese and aged mice. Non‐exercising mice (open circles) were used as controls for running mice (filled circles). Results are means ± SD (*n *= 3–6). *Significant difference versus corresponding non‐exercising controls; ^a^significant difference versus baseline (‘B’); *P *≤ 0.05 (two‐way ANOVA). Abbreviation: FGF21, fibroblast growth factor 21.

**FIGURE A7:**
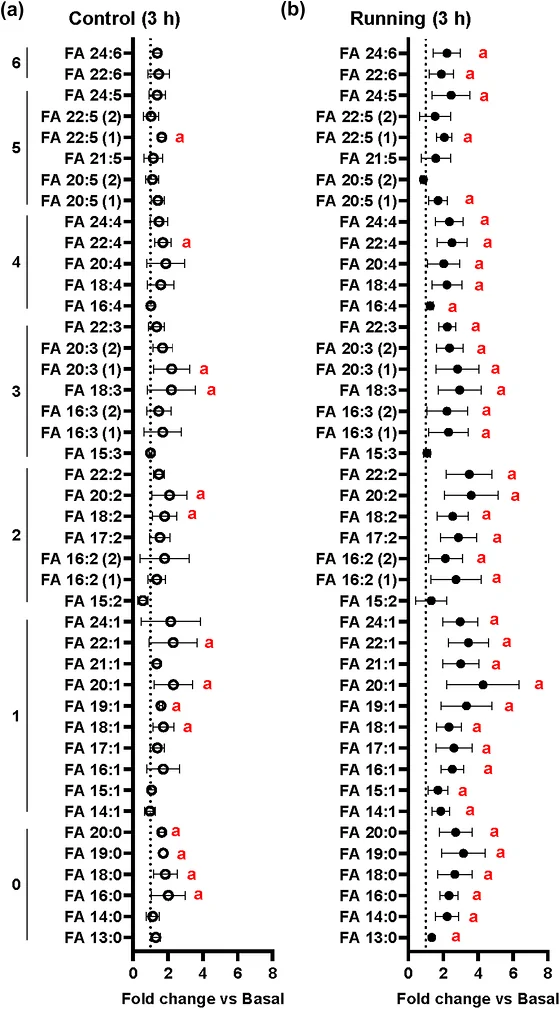
Relative changes in fatty acid concentrations in epididymal AT of lean mice after exercise. Changes in the concentration of various fatty acids in the epididymal AT of control and running mice were measured at the 3 h time point. Results are means ± SD (*n *= 4–6) and are expressed as a fold change from the baseline (‘Basal’). ^a^Significant difference versus baseline; *P *≤ 0.05 (Student's unpaired *t*‐test). Abbreviations: AT, adipose tissue; FA, fatty acid.

## Data Availability

The datasets analysed during the present study are available from the corresponding author on reasonable request.

## Liquid chromatography–mass spectrometry analysis

Lipidomic profiling of epididymal AT samples was performed using an untargeted workflow for the lipidome, metabolome and exposome analysis, as reported before (Janovska et al., 2020; Sistilli et al., 2021). Briefly, AT samples were extracted using a biphasic solvent system of cold methanol, methyl *tert*‐butyl ether (MTBE) and 10% methanol. Then, LC‐MS‐based lipidomic profiling of high‐abundant TGs in positive ion mode and minor polar lipids in positive and negative ion modes was performed using the reversed‐phase LC mechanism. Hydrophilic interaction chromatography (HILIC) in positive mode was used to analyse short‐chain acylcarnitines. The LC‐MS systems consisted of a Vanquish UHPLC system (Thermo Fisher Scientific), an OptaMax NG ion source with a HESI‐II probe (Thermo Fisher Scientific) and an Orbitrap Exploris 480 mass spectrometer (Thermo Fisher Scientific).

The YMC‐Triart C18 ExRS column (150 mm × 2 mm i.d.; 1.9 µm particle size; YMC America, Devens, MA, USA) equipped with a holder‐cartridge assembly with C18 guard cartridge (Phenomenex) was used to separate FAHFA species. The separation was carried out at a flow rate of 0.2 mL/min, with the column kept at a temperature of 50°C. The mobile phase included 70:30 water/acetonitrile with 0.1% acetic acid (A) and 50:50 isopropanol/acetonitrile with 0.1% acetic acid (B). Separation was achieved through the following gradient: 0–1 min 0% (B); 1–5 min from 0% to 90% (B); 5–23 min 90% (B), 23–24 min from 90% to 100% (B), 24–26 min 100% (B), 26–28 min from 100% to 0% (B), and 28–31 min 0% (B) + 1 min pre‐injection steps. The injection volume was 10 µL. The sample temperature was kept at 4°C.

The ion source parameters were as follows: sheath gas pressure, 45 a.u.; aux gas flow, 10 a.u.; sweep gas flow, 2 a.u.; ion transfer tube temperature, 300°C; vaporizer temperature, 400°C; and spray voltage, −2.5 kV. The mass spectrometer settings were set to MA1 mass range, *m*/z 505–610; MA1 resolving power, 90 000 FWHM (*m*/*z* 200); the number of data‐dependent scans per cycle, 5; and MS/MS resolving power, 15 000 FWHM (*m*/*z* 200). For MS/MS experiments, normalized collision energies of 20% and 30% were set up.

Samples were randomized within the sequence with regular injection of quality control (QC) pool samples at the beginning, end and between every 10 samples. The sequence also included method blanks and serial dilution samples prepared from QC sample (0, 1/16, 1/8, 1/4, 1/2 and 1).

The LC‐MS instrumental files were processed using MS‐DIAL v.4.9.221218 software (Tsugawa et al., 2020), including annotation of FAHFA using MA1 and MS/MS spectra. FAHFA were reported at the species level as the total number of carbon atoms and double bonds (e.g., FAHFA 34:0;O) and at the molecular species level (FAHFA 16:0/18:0;O). Exported data sets as signal intensities from the detector (peak heights) were filtered further by method blanks, a dilution series of QC samples, and QC samples (a relative standard deviation > 30%). Data were then normalized using locally estimated scatterplot smoothing (LOESS), the amount taken for the analysis, and using [^13^C4]‐9‐PAHSA internal standard. The subsequent regioisomer annotation of FAHFA was based on the previous run of authentic standards (Riecan et al., 2024).

## FAHFA analysis

Samples of AT (40–50 mg) and plasma (120 µL) were extracted according to previously described methods, with several modifications to improve extraction yield and FAHFA detection during LC‐MS analysis (Riecan et al., 2024; Yore et al., 2014). Samples were homogenized using a ball mill (see section 2.8) in a mixture of citric acid buffer and ethyl acetate (ratio 1:2, v/v) containing 1 ng of [^13^C4]‐9‐PAHSA (Cayman Chemical; Ann Arbor, MI, USA) as an internal standard. The ethyl acetate phase was collected and dried using a Savant SpeedVac (Thermo Fisher Scientific, Bremen, Germany). Based on optimization of extraction yield, we dissolved the dried extracts in 300 µL (200 µL for plasma) of chloroform instead of a mixture of ethyl acetate and hexane (5:95, v/v) and loaded the samples onto HyperSep solid‐phase extraction columns (500 mg/10 mL, 40–60 µm, 70 Å; Thermo Scientific) for further purification by solid‐phase extraction. FAHFA were eluted with 5 mL ethyl acetate and dried. Before analysis, purified extracts were dissolved in 50 µL of a methanol/water mixture (95:5, v/v) to eliminate TG, potentially suppressing the FAHFA signal. Samples were analysed by LC‐MS as before (Riecan et al., 2024).

**TABLE A1:** Gene names, gene symbols, and sequences of the oligonucleotide primers.

Name	Abbreviation	5′ sequence	3′ sequence
Acetyl‐CoA carboxylase 1	*Acc1*	AGCAGATCCGCAGCTTGGTCC	AGATGGGAGAGGCAGCCCGA
Acetyl‐CoA carboxylase 2	*Acc2*	TGCGCTCCACCATCCGTGAAAACAT	TTCCCAAAATAAGCCCGTGTCC
ATP citrate synthase	*Acly*	GTGGCGGGGAAGTGCTGTTTGA	TGTGCTCGGGCTGGGAAGGAC
Androgen dependent TFPI regulating protein	*Adtrp*	CCAGGGAAGCCATGACGAAAACA	GGCAGGCGACCCCAAAGAAAA
Androgen induced 1	*Aig1*	AGAAGGTGCATATGGCGGCGAGTC	TGTTTGTTGTTGCGGTGTTCTGGA
Adipose triglyceride lipase	*Atgl*	GGCAATCAGCAGGCAGGGTCTTTA	GCCAACGCCACTCACATCTACG
Abhydrolase Domain Containing 5, Lysophosphatidic Acid Acyltransferase	*Cgi‐58*	ATGCTTCAGCGGATAGGTGG	GCATACACATAATGCCCCGC
Carbohydrate‐responsive element‐binding protein	*ChREBP*	TGCCACTGCCAGAGACAACAACC	ACGGGCACGGGGGATTTCA
Eukaryotic translation elongation factor 2	*Eef2*	GAAACGCGCAGATGTCCAAAAGTC	GCCGGGCTGCAAGTCTAAGG
ELOVL fatty acid elongase 5	*Elovl5*	CCTCTCGGGTGGCTGTTCTTCC	AGGCTTCGGCTCGGCTTGTC
Fatty acid synthase	*Fasn*	TGGGTGTGGAAGTTCGTCAG	GTCGTGTCAGTAGCCGAGTC
Glucose transporter type 4	*Glut4*	CAGTATGTTGCGGATGCTATGG	TTCTCATCTGGCCCTAAGTATTCA
Glutathione peroxidase 1	*Gpx1*	GGTTTCCCGTGCAATCAGTTCG	GAGCGCAGTGGGGTCGTCA
Hormone‐sensitive lipase	*Hsl*	TGCGCCCCACGGAGTCTATGC	CTCGGGGCTGTCTGAAGGCTCTGA
Microsomal glutathione S‐transferase 1	*Mgst1*	CCTGTTTGGCTGAGGAAGGGGA	TGCGCAGAGCCCACCTGAATGA
Nuclear factor erythroid 2‐related factor 2	*Nrf2*	AAGGCTCCATCCTCCCGAACC	CTGAAAAGGCGGCTCAGCACC
Peroxiredoxin 6	*Prdx6*	TCGGAGAGGGTGGGAACTACC	CAGTTGACTGGAAGAAGGGAGAGAG
Stearoyl‐CoA desaturase 1	*Scd1*	TGGCTAAGAGGCACCTTGGCTG	TGGCACTGGGGCTGCTAATCT
Sterol regulatory element binding transcription factor 1	*Srebf1*	TACCCGTCCGTGTCCCCCTTTTC	TGCGCTTCTCACCACGGCTCTG

**TABLE A2:** List of annotated complex lipids in epididymal adipose tissue samples.

Metabolite name (full)	Metabolite name (short)	Subclass	Average retention time (min)	Average *m/z*	Platform
CAR 2:0; [M+H]+	CAR 2:0	CAR	3.40	204.1228	HILICp
CAR 3:0; [M+H]+	CAR 3:0	CAR	2.99	218.1384	HILICp
CAR 4:0; [M+H]+	CAR 4:0	CAR	2.65	232.1541	HILICp
CAR 4:0‐OH; [M+H]+	CAR 4:0‐OH	CAR	3.67	248.1489	HILICp
CAR 5:0; [M+H]+	CAR 5:0	CAR	2.42	246.1697	HILICp
CAR 5:0‐OH; [M+H]+	CAR 5:0‐OH	CAR	3.46	262.1643	HILICp
CAR 6:0; [M+H]+	CAR 6:0	CAR	2.20	260.1851	HILICp
CAR 10:0; [M+H]+	CAR 10:0	CAR	0.29	316.2485	LIPp
CAR 12:0; [M+H]+	CAR 12:0	CAR	1.38	344.2796	HILICp
CAR 14:0; [M+H]+	CAR 14:0	CAR	0.54	372.3105	LIPp
CAR 14:1; [M+H]+	CAR 14:1	CAR	0.42	370.2951	LIPp
CAR 16:0; [M+H]+	CAR 16:0	CAR	0.83	400.3421	LIPp
CAR 16:1; [M+H]+	CAR 16:1	CAR	0.60	398.3263	LIPp
CAR 17:0; [M+H]+	CAR 17:0	CAR	0.97	414.3576	LIPp
CAR 18:0; [M+H]+	CAR 18:0	CAR	1.26	428.3736	LIPp
CAR 18:1; [M+H]+	CAR 18:1	CAR	0.91	426.3578	LIPp
CAR 18:2; [M+H]+	CAR 18:2	CAR	0.68	424.3416	LIPp
CAR 20:1; [M+H]+	CAR 20:1	CAR	1.32	454.3895	LIPp
CAR 20:4; [M+H]+	CAR 20:4	CAR	0.66	448.3418	LIPp
Cer 34:1;2O; Cer 18:1;2O/16:0; [M+H]+	Cer 34:1;2O	Cer	3.01	538.5200	LIPp
Cer 34:2;2O; Cer 18:2;2O/16:0; [M+H]+	Cer 34:2;2O	Cer	2.72	536.5041	LIPp
Cer 36:2;2O; Cer 18:2;2O/18:0; [M+H]+	Cer 36:2;2O	Cer	3.04	564.5358	LIPp
Cer 38:2;2O; Cer 18:2;2O/20:0; [M+H]+	Cer 38:2;2O	Cer‐NS	3.38	592.5659	LIPp
Cer 40:2;2O; Cer 18:2;2O/22:0; [M+H]+	Cer 40:2;2O	Cer‐NS	3.70	620.5983	LIPp
Cer 42:3;2O; Cer 18:2;2O/24:1; [M+H]+	Cer 42:3;2O	Cer	3.69	646.6138	LIPp
Cholesterol; [M‐H2O+H]+	Cholesterol	Cholesterol	2.54	369.3515	LIPp
DG 32:1; DG 16:0_16:1; [M+NH4]+	DG 32:1	DG	3.29	584.5250	LIPp
DG 32:2; DG 14:0_18:2; [M+NH4]+	DG 32:2	DG	3.01	582.5084	LIPp
DG 34:1; DG 16:0_18:1; [M+NH4]+	DG 34:1	DG	3.61	612.5562	LIPp
DG 34:2; DG 16:0_18:2; [M+NH4]+	DG 34:2	DG	3.34	610.5405	LIPp
DG 34:3; DG 16:1_18:2; [M+NH4]+	DG 34:3	DG	3.05	608.5247	LIPp
DG 35:2; DG 17:1_18:1; [M+NH4]+	DG 35:2	DG	3.47	624.5567	LIPp
DG 36:1; DG 18:0_18:1; [M+NH4]+	DG 36:1	DG	3.92	640.5881	LIPp
DG 36:2; DG 18:1_18:1; [M+NH4]+	DG 36:2	DG	3.63	638.5721	LIPp
DG 36:3; DG 18:1_18:2; [M+NH4]+	DG 36:3	DG	3.36	636.5560	LIPp
DG 36:4; DG 18:2_18:2; [M+NH4]+	DG 36:4	DG	3.10	634.5400	LIPp
DG 36:5; DG 18:2_18:3; [M+NH4]+	DG 36:5	DG	2.87	632.5247	LIPp
DG 38:2; DG 18:1_20:1; [M+NH4]+	DG 38:2	DG	3.92	666.6032	LIPp
DG 38:3; DG 20:1_18:2; [M+NH4]+	DG 38:3	DG	3.66	664.5845	LIPp
DG 38:5; DG 18:1_20:4; [M+NH4]+	DG 38:5	DG	3.28	660.5564	LIPp
FA 13:0; [M‐H]‐	FA 13:0	FA	0.70	213.1859	LIPn
FA 14:0; [M‐H]‐	FA 14:0	FA	1.10	227.2013	LIPn
FA 14:1; [M‐H]‐	FA 14:1	FA	0.80	225.1857	LIPn
FA 15:1; [M‐H]‐	FA 15:1	FA	1.02	239.2012	LIPn
FA 15:2; [M‐H]‐	FA 15:2	FA	0.70	237.1856	LIPn
FA 15:3; [M‐H]‐	FA 15:3	FA	0.69	235.1700	LIPn
FA 16:0; [M‐H]‐	FA 16:0	FA	1.60	255.2326	LIPn
FA 16:1; [M‐H]‐	FA 16:1	FA	1.20	253.2168	LIPn
FA 16:2 (1); [M‐H]‐	FA 16:2 (1)	FA	0.92	251.2014	LIPn
FA 16:2 (2); [M‐H]‐	FA 16:2 (2)	FA	1.01	251.2014	LIPn
FA 16:3 (1); [M‐H]‐	FA 16:3 (1)	FA	0.70	249.1856	LIPn
FA 16:3 (2); [M‐H]‐	FA 16:3 (2)	FA	0.76	249.1858	LIPn
FA 16:4; [M‐H]‐	FA 16:4	FA	0.53	247.1701	LIPn
FA 17:1; [M‐H]‐	FA 17:1	FA	1.44	267.2325	LIPn
FA 17:2; [M‐H]‐	FA 17:2	FA	1.11	265.2168	LIPn
FA 18:0; [M‐H]‐	FA 18:0	FA	1.92	283.2637	LIPn
FA 18:1; [M‐H]‐	FA 18:1	FA	1.67	281.2479	LIPn
FA 18:2; [M‐H]‐	FA 18:2	FA	1.33	279.2323	LIPn
FA 18:3; [M‐H]‐	FA 18:3	FA	1.04	277.2167	LIPn
FA 18:4; [M‐H]‐	FA 18:4	FA	0.83	275.2013	LIPn
FA 19:0; [M‐H]‐	FA 19:0	FA	2.05	297.2798	LIPn
FA 19:1; [M‐H]‐	FA 19:1	FA	1.82	295.2638	LIPn
FA 20:0; [M‐H]‐	FA 20:0	FA	2.20	311.2956	LIPn
FA 20:1; [M‐H]‐	FA 20:1	FA	1.94	309.2795	LIPn
FA 20:2; [M‐H]‐	FA 20:2	FA	1.74	307.2638	LIPn
FA 20:3 (1); [M‐H]‐	FA 20:3 (1)	FA	1.49	305.2482	LIPn
FA 20:3 (2); [M‐H]‐	FA 20:3 (2)	FA	1.59	305.2480	LIPn
FA 20:4; [M‐H]‐	FA 20:4	FA	1.26	303.2325	LIPn
FA 20:5 (1); [M‐H]‐	FA 20:5 (1)	FA	0.99	301.2170	LIPn
FA 20:5 (2); [M‐H]‐	FA 20:5 (2)	FA	1.17	301.2168	LIPn
FA 21:1; [M‐H]‐	FA 21:1	FA	2.07	323.2956	LIPn
FA 21:5; [M‐H]‐	FA 21:5	FA	1.16	315.2322	LIPn
FA 22:1; [M‐H]‐	FA 22:1	FA	2.21	337.3111	LIPn
FA 22:2; [M‐H]‐	FA 22:2	FA	2.00	335.2956	LIPn
FA 22:3; [M‐H]‐	FA 22:3	FA	1.84	333.2799	LIPn
FA 22:4; [M‐H]‐	FA 22:4	FA	1.63	331.2638	LIPn
FA 22:5 (1); [M‐H]‐	FA 22:5 (1)	FA	1.34	329.2480	LIPn
FA 22:5 (2); [M‐H]‐	FA 22:5 (2)	FA	1.45	329.2483	LIPn
FA 22:6; [M‐H]‐	FA 22:6	FA	1.16	327.2326	LIPn
FA 24:1; [M‐H]‐	FA 24:1	FA	2.52	365.3422	LIPn
FA 24:4; [M‐H]‐	FA 24:4	FA	1.88	359.2954	LIPn
FA 24:5; [M‐H]‐	FA 24:5	FA	1.75	357.2796	LIPn
FA 24:6; [M‐H]‐	FA 24:6	FA	1.52	355.2637	LIPn
LPC 16:0/0:0; [M+H]+	LPC 16:0/0:0	LPC	0.79	496.3397	LIPp
LPC 16:1/0:0; [M+H]+	LPC 16:1/0:0	LPC	0.56	494.3241	LIPp
LPC 17:0/0:0; [M+H]+	LPC 17:0/0:0	LPC	0.98	510.3556	LIPp
LPC 18:0/0:0; [M+H]+	LPC 18:0/0:0	LPC	1.21	524.3712	LIPp
LPC 18:1/0:0; [M+H]+	LPC 18:1/0:0	LPC	0.86	522.3557	LIPp
LPC 18:2/0:0; [M+H]+	LPC 18:2/0:0	LPC	0.64	520.3399	LIPp
LPC 19:0/0:0; [M+H]+	LPC 19:0/0:0	LPC	1.46	538.3873	LIPp
LPC 20:0/0:0; [M+H]+	LPC 20:0/0:0	LPC	1.68	552.4029	LIPp
LPC 20:1/0:0; [M+H]+	LPC 20:1/0:0	LPC	1.28	550.3871	LIPp
LPC 20:2/0:0; [M+H]+	LPC 20:2/0:0	LPC	0.96	548.3715	LIPp
LPC 20:3/0:0; [M+H]+	LPC 20:3/0:0	LPC	0.74	546.3557	LIPp
LPC 20:4/0:0; [M+H]+	LPC 20:4/0:0	LPC	0.58	544.3396	LIPp
LPC 20:5/0:0; [M+H]+	LPC 20:5/0:0	LPC	0.64	542.3220	LIPp
LPC 22:0/0:0; [M+H]+	LPC 22:0/0:0	LPC	1.96	580.4338	LIPp
LPC 22:6/0:0; [M+H]+	LPC 22:6/0:0	LPC	0.56	568.3397	LIPp
LPC 24:0/0:0; [M+H]+	LPC 24:0/0:0	LPC	2.24	608.4651	LIPp
LPC 24:1/0:0; [M+H]+	LPC 24:1/0:0	LPC	1.97	606.4491	LIPp
LPE 16:0; [M‐H]‐	LPE 16:0	LPE	0.81	452.2780	LIPn
LPE 16:1; [M‐H]‐	LPE 16:1	LPE	0.57	450.2627	LIPn
LPE 18:0; [M‐H]‐	LPE 18:0	LPE	1.25	480.3079	LIPn
LPE 18:1; [M‐H]‐	LPE 18:1	LPE	0.89	478.2935	LIPn
LPE 18:2; [M‐H]‐	LPE 18:2	LPE	0.61	476.2776	LIPn
LPE 20:4; [M‐H]‐	LPE 20:4	LPE	0.59	500.2776	LIPn
LPE 22:6; [M‐H]‐	LPE 22:6	LPE	0.57	524.2778	LIPn
LPE O‐16:1; [M‐H]‐	LPE O‐16:1	EtherLPE	0.93	436.2829	LIPn
MG 16:1; [M+Na]+	MG 16:1	MG	1.04	351.2505	LIPp
MG 18:1 (1); [M+Na]+	MG 18:1 (1)	MG	1.44	379.2819	LIPp
MG 18:1 (2); [M+Na]+	MG 18:1 (2)	MG	1.51	379.2817	LIPp
MG 18:2 (1); [M+Na]+	MG 18:2 (1)	MG	1.10	377.2662	LIPp
MG 18:2 (2); [M+Na]+	MG 18:2 (2)	MG	1.16	377.2662	LIPp
NAE 16:0; [M+H]+	NAE 16:0	NAE	1.15	300.2898	LIPp
NAE 18:0; [M+H]+	NAE 18:0	NAE	1.64	328.3210	LIPp
NAE 18:1 (1); [M+H]+	NAE 18:1 (1)	NAE	1.05	326.3054	LIPp
NAE 18:1 (2); [M+H]+	NAE 18:1 (2)	NAE	1.25	326.3055	LIPp
NAE 18:2; [M+H]+	NAE 18:2	NAE	0.94	324.2896	LIPp
NAE 20:1; [M+H]+	NAE 20:1	NAE	1.69	354.3369	LIPp
PC 30:0; PC 14:0_16:0; [M+H]+	PC 30:0	PC	2.66	706.5366	LIPp
PC 30:1; [M+H]+	PC 30:1	PC	2.41	704.5223	LIPp
PC 31:0; [M+H]+	PC 31:0	PC	2.82	720.5527	LIPp
PC 32:0; PC 16:0_16:0; [M+H]+	PC 32:0	PC	2.99	734.5691	LIPp
PC 32:1; [M+H]+	PC 32:1	PC	2.70	732.5533	LIPp
PC 32:2; PC 16:1_16:1; [M+H]+	PC 32:2	PC	2.45	730.5383	LIPp
PC 33:1; [M+H]+	PC 33:1	PC	2.85	746.5692	LIPp
PC 33:2; [M+H]+	PC 33:2	PC	2.61	744.5529	LIPp
PC 34:1; PC 16:0_18:1; [M+H]+	PC 34:1	PC	3.01	760.5846	LIPp
PC 34:2; PC 16:0_18:2; [M+H]+	PC 34:2	PC	2.76	758.5689	LIPp
PC 34:3 (1); [M+H]+	PC 34:3 (1)	PC	2.50	756.5537	LIPp
PC 34:3 (2); [M+H]+	PC 34:3 (2)	PC	2.60	756.5535	LIPp
PC 34:3 (3); PC 16:0_18:3; [M+H]+	PC 34:3 (3)	PC	2.98	756.5507	LIPp
PC 34:4; [M+H]+	PC 34:4	PC	2.41	754.5382	LIPp
PC 35:1; [M+H]+	PC 35:1	PC	3.16	774.6003	LIPp
PC 35:2; PC 17:0_18:2; [M+H]+	PC 35:2	PC	2.91	772.5850	LIPp
PC 35:3; [M+H]+	PC 35:3	PC	2.64	770.5699	LIPp
PC 35:4; [M+H]+	PC 35:4	PC	2.56	768.5548	LIPp
PC 36:1; PC 18:0_18:1; [M+H]+	PC 36:1	PC	3.33	788.6165	LIPp
PC 36:2; PC 18:0_18:2; [M+H]+	PC 36:2	PC	3.07	786.6003	LIPp
PC 36:3 (1); [M+H]+	PC 36:3 (1)	PC	2.23	784.5854	LIPp
PC 36:3 (2); [M+H]+	PC 36:3 (2)	PC	2.78	784.5845	LIPp
PC 36:4 (1); PC 18:2_18:2; [M+H]+	PC 36:4 (1)	PC	2.55	782.5689	LIPp
PC 36:4 (2); PC 16:0_20:4; [M+H]+	PC 36:4 (2)	PC	2.71	782.5689	LIPp
PC 36:5 (1); [M+H]+	PC 36:5 (1)	PC	2.35	780.5542	LIPp
PC 36:5 (2); [M+H]+	PC 36:5 (2)	PC	2.45	780.5538	LIPp
PC 36:5 (3); [M+H]+	PC 36:5 (3)	PC	2.51	780.5530	LIPp
PC 36:5 (4); PC 16:0_20:5; [M+H]+	PC 36:5 (4)	PC	2.76	780.5505	LIPp
PC 37:1; [M+H]+	PC 37:1	PC	3.48	802.6309	LIPp
PC 37:2; [M+H]+	PC 37:2	PC	3.23	800.6163	LIPp
PC 37:3; [M+H]+	PC 37:3	PC	2.92	798.6001	LIPp
PC 37:4; [M+H]+	PC 37:4	PC	2.86	796.5843	LIPp
PC 38:2; [M+H]+	PC 38:2	PC	3.35	814.6312	LIPp
PC 38:3 (1); [M+H]+	PC 38:3 (1)	PC	3.07	812.6153	LIPp
PC 38:3 (2); [M+H]+	PC 38:3 (2)	PC	3.15	812.6161	LIPp
PC 38:4 (1); [M+H]+	PC 38:4 (1)	PC	2.85	810.6001	LIPp
PC 38:4 (2); [M+H]+	PC 38:4 (2)	PC	2.92	810.6000	LIPp
PC 38:4 (3); PC 18:0_20:4; [M+H]+	PC 38:4 (3)	PC	3.02	810.5998	LIPp
PC 38:4 (4); [M+H]+	PC 38:4 (4)	PC	3.33	810.5980	LIPp
PC 38:5 (1); [M+H]+	PC 38:5 (1)	PC	2.72	808.5847	LIPp
PC 38:5 (2); PC 18:0_20:5; [M+H]+	PC 38:5 (2)	PC	3.07	808.5819	LIPp
PC 38:6 (1); [M+H]+	PC 38:6 (1)	PC	2.50	806.5689	LIPp
PC 38:6 (2); PC 16:0_22:6; [M+H]+	PC 38:6 (2)	PC	2.62	806.5688	LIPp
PC 38:7; [M+H]+	PC 38:7	PC	2.37	804.5541	LIPp
PC 39:4; [M+H]+	PC 39:4	PC	3.17	824.6156	LIPp
PC 40:4; [M+H]+	PC 40:4	PC	3.24	838.6320	LIPp
PC 40:5 (1); [M+H]+	PC 40:5 (1)	PC	3.02	836.6157	LIPp
PC 40:5 (2); [M+H]+	PC 40:5 (2)	PC	3.14	836.6138	LIPp
PC 40:6 (1); [M+H]+	PC 40:6 (1)	PC	2.75	834.6001	LIPp
PC 40:6 (2); [M+H]+	PC 40:6 (2)	PC	2.92	834.6003	LIPp
PC 40:7; [M+H]+	PC 40:7	PC	2.64	832.5841	LIPp
PC 40:8; [M+H]+	PC 40:8	PC	2.43	830.5693	LIPp
PC 42:10; [M+H]+	PC 42:10	PC	2.36	854.5700	LIPp
PC O‐34:1; [M+H]+	PC O‐34:1	EtherPC	3.19	746.6071	LIPp
PC O‐34:2; [M+H]+	PC O‐34:2	EtherPC	2.94	744.5899	LIPp
PC O‐35:8; [M+H]+	PC O‐35:8	EtherPC	2.92	746.5088	LIPp
PC O‐36:1; [M+H]+	PC O‐36:1	EtherPC	3.52	774.6384	LIPp
PC O‐36:3; [M+H]+	PC O‐36:3	EtherPC	2.95	770.6072	LIPp
PC O‐36:4; [M+H]+	PC O‐36:4	EtherPC	2.88	768.5907	LIPp
PC O‐36:5; [M+H]+	PC O‐36:5	EtherPC	2.83	766.5773	LIPp
PC O‐37:8; [M+H]+	PC O‐37:8	EtherPC	3.24	774.5407	LIPp
PC O‐38:4; [M+H]+	PC O‐38:4	EtherPC	3.20	796.6235	LIPp
PC O‐38:5; [M+H]+	PC O‐38:5	EtherPC	2.90	794.6073	LIPp
PC O‐38:6; [M+H]+	PC O‐38:6	EtherPC	2.79	792.5903	LIPp
PC O‐38:7; [M+H]+	PC O‐38:7	EtherPC	2.74	790.5718	LIPp
PC O‐40:4; [M+H]+	PC O‐40:4	EtherPC	3.37	824.6517	LIPp
PC O‐40:5; [M+H]+	PC O‐40:5	EtherPC	3.12	822.6376	LIPp
PC O‐40:6; [M+H]+	PC O‐40:6	EtherPC	2.90	820.6232	LIPp
PC O‐40:7; [M+H]+	PC O‐40:7	EtherPC	2.80	818.6063	LIPp
PC 34:2;2O; PC 16:0_18:2;2O; [M+CH3COO]‐	PC 34:2;2O	OxPC	2.02	848.5653	LIPn
PC 36:2;2O; PC 18:0_18:2;2O; [M+CH3COO]‐	PC 36:2;2O	OxPC	2.24	876.5966	LIPn
PE 32:1; PE 16:0_16:1; [M‐H]‐	PE 32:1	PE	2.78	688.4910	LIPn
PE 32:2; PE 16:1_16:1; [M‐H]‐	PE 32:2	PE	2.51	686.4760	LIPn
PE 34:1; PE 16:0_18:1; [M‐H]‐	PE 34:1	PE	3.10	716.5227	LIPn
PE 34:2; PE 16:0_18:2; [M‐H]‐	PE 34:2	PE	2.84	714.5068	LIPn
PE 34:3; PE 16:1_18:2; [M‐H]‐	PE 34:3	PE	2.57	712.4912	LIPn
PE 36:1; PE 18:0_18:1; [M‐H]‐	PE 36:1	PE	3.42	744.5549	LIPn
PE 36:2; PE 18:0_18:2; [M‐H]‐	PE 36:2	PE	3.16	742.5385	LIPn
PE 36:3; PE 18:1_18:2; [M‐H]‐	PE 36:3	PE	2.87	740.5226	LIPn
PE 36:4 (1); PE 18:2_18:2; [M‐H]‐	PE 36:4 (1)	PE	2.62	738.5066	LIPn
PE 36:4 (2); PE 16:0_20:4; [M‐H]‐	PE 36:4 (2)	PE	2.79	738.5067	LIPn
PE 36:5; PE 16:1_20:4; [M‐H]‐	PE 36:5	PE	2.51	736.4915	LIPn
PE 38:3; PE 18:0_20:3; [M‐H]‐	PE 38:3	PE	3.24	768.5543	LIPn
PE 38:4; PE 18:0_20:4; [M‐H]‐	PE 38:4	PE	3.11	766.5383	LIPn
PE 38:5; PE 18:1_20:4; [M‐H]‐	PE 38:5	PE	2.81	764.5225	LIPn
PE 38:6 (1); PE 18:2_20:4; [M‐H]‐	PE 38:6 (1)	PE	2.57	762.5069	LIPn
PE 38:6 (2); PE 16:0_22:6; [M‐H]‐	PE 38:6 (2)	PE	2.70	762.5077	LIPn
PE 38:7; PE 16:1_22:6; [M‐H]‐	PE 38:7	PE	2.44	760.4916	LIPn
PE 40:4; PE 18:0_22:4; [M‐H]‐	PE 40:4	PE	3.33	794.5694	LIPn
PE 40:5 (1); PE 18:0_22:5; [M‐H]‐	PE 40:5 (1)	PE	3.11	792.5537	LIPn
PE 40:5 (2); PE 18:0_22:5; [M‐H]‐	PE 40:5 (2)	PE	3.23	792.5541	LIPn
PE 40:6 (1); PE 18:1_22:5; [M‐H]‐	PE 40:6 (1)	PE	2.93	790.5406	LIPn
PE 40:6 (2); PE 18:0_22:6; [M‐H]‐	PE 40:6 (2)	PE	3.01	790.5390	LIPn
PE 40:7; PE 18:1_22:6; [M‐H]‐	PE 40:7	PE	2.72	788.5231	LIPn
PE 40:8; PE 20:4_20:4; [M‐H]‐	PE 40:8	PE	2.49	786.5082	LIPn
PE O‐32:2; PE O‐16:1_16:1; [M‐H]‐	PE O‐32:2	EtherPE	2.93	672.4965	LIPn
PE O‐34:2; PE O‐16:1_18:1; [M‐H]‐	PE O‐34:2	EtherPE	3.25	700.5279	LIPn
PE O‐34:3; PE O‐16:1_18:2; [M‐H]‐	PE O‐34:3	EtherPE	2.99	698.5121	LIPn
PE O‐36:3; PE O‐18:1_18:2; [M‐H]‐	PE O‐36:3	EtherPE	3.30	726.5437	LIPn
PE O‐36:5; PE O‐16:1_20:4; [M‐H]‐	PE O‐36:5	EtherPE	2.93	722.5123	LIPn
PE O‐38:5 (1); PE O‐16:1_22:4; [M‐H]‐	PE O‐38:5 (1)	EtherPE	3.16	750.5433	LIPn
PE O‐38:5 (2); PE O‐18:1_20:4; [M‐H]‐	PE O‐38:5 (2)	EtherPE	3.25	750.5435	LIPn
PE O‐38:6 (1); PE O‐18:2_20:4; [M‐H]‐	PE O‐38:6 (1)	EtherPE	2.94	748.5324	LIPn
PE O‐38:6 (2); PE O‐16:1_22:5; [M‐H]‐	PE O‐38:6 (2)	EtherPE	3.05	748.5272	LIPn
PE O‐38:7 (1); PE O‐18:3_20:4; [M‐H]‐	PE O‐38:7 (1)	EtherPE	2.70	746.5118	LIPn
PE O‐38:7 (2); PE O‐16:1_22:6; [M‐H]‐	PE O‐38:7 (2)	EtherPE	2.83	746.5123	LIPn
PE O‐40:7; PE O‐18:1_22:6; [M‐H]‐	PE O‐40:7	EtherPE	3.14	774.5435	LIPn
PE O‐40:8; PE O‐18:2_22:6; [M‐H]‐	PE O‐40:8	EtherPE	2.85	772.5277	LIPn
PE 36:3;2O; PE 18:1_18:2;2O; [M‐H]‐	PE 36:3;2O	OxPE	2.08	772.5127	LIPn
PE 40:7;O; PE 20:4_20:3;O; [M‐H]‐	PE 40:7;O	OxPE	2.92	804.5159	LIPn
PEtOH 34:1; PEtOH 16:0_18:1; [M‐H]‐	PEtOH 34:1	PEtOH	2.89	701.5115	LIPn
PEtOH 34:2; PEtOH 16:0_18:2; [M‐H]‐	PEtOH 34:2	PEtOH	2.66	699.4962	LIPn
PEtOH 36:2; PEtOH 18:0_18:2; [M‐H]‐	PEtOH 36:2	PEtOH	2.95	727.5279	LIPn
PEtOH 36:3; PEtOH 18:1_18:2; [M‐H]‐	PEtOH 36:3	PEtOH	2.68	725.5118	LIPn
PEtOH 36:4; PEtOH 16:0_20:4; [M‐H]‐	PEtOH 36:4	PEtOH	2.61	723.4963	LIPn
PEtOH 38:5; PEtOH 18:1_20:4; [M‐H]‐	PEtOH 38:5	PEtOH	2.63	749.5114	LIPn
PG 34:1 (1); PG 16:0_18:1; [M‐H]‐	PG 34:1 (1)	PG	2.51	747.5108	LIPn
PG 34:1 (2); PG 16:0_18:1; [M‐H]‐	PG 34:1 (2)	PG	2.71	747.5171	LIPn
PG 34:2 (1); PG 16:1_18:1; [M‐H]‐	PG 34:2 (1)	PG	2.29	745.5029	LIPn
PG 34:2 (2); PG 16:0_18:2; [M‐H]‐	PG 34:2 (2)	PG	2.50	745.5015	LIPn
PG 36:2; PG 18:1_18:1; [M‐H]‐	PG 36:2	PG	2.53	773.5330	LIPn
PG 36:3; PG 18:1_18:2; [M‐H]‐	PG 36:3	PG	2.34	771.5176	LIPn
PG 36:4; PG 18:2_18:2; [M‐H]‐	PG 36:4	PG	2.16	769.5028	LIPn
PG 38:5; PG 18:1_20:4; [M‐H]‐	PG 38:5	PG	2.30	795.5179	LIPn
PG 38:6; PG 18:2_20:4; [M‐H]‐	PG 38:6	PG	2.13	793.5023	LIPn
PG 40:7; PG 18:1_22:6; [M‐H]‐	PG 40:7	PG	2.24	819.5177	LIPn
PI 34:2; [M‐H]‐	PI 34:2	PI	2.43	833.5177	LIPn
PI 36:2; PI 18:1_18:1; [M‐H]‐	PI 36:2	PI	2.69	861.5487	LIPn
PI 36:3; PI 18:1_18:2; [M‐H]‐	PI 36:3	PI	2.46	859.5333	LIPn
PI 36:4; PI 16:0_20:4; [M‐H]‐	PI 36:4	PI	2.39	857.5178	LIPn
PI 37:4; [M‐H]‐	PI 37:4	PI	2.53	871.5325	LIPn
PI 38:3; PI 18:0_20:3; [M‐H]‐	PI 38:3	PI	2.88	887.5638	LIPn
PI 38:4; PI 18:0_20:4; [M‐H]‐	PI 38:4	PI	2.67	885.5486	LIPn
PI 38:5; PI 18:1_20:4; [M‐H]‐	PI 38:5	PI	2.42	883.5333	LIPn
PS 34:2; PS 16:0_18:2; [M‐H]‐	PS 34:2	PS	2.46	758.4973	LIPn
PS 36:1; PS 18:0_18:1; [M‐H]‐	PS 36:1	PS	2.98	788.5437	LIPn
PS 36:2; PS 18:0_18:2; [M‐H]‐	PS 36:2	PS	2.74	786.5281	LIPn
PS 36:3; PS 18:1_18:2; [M‐H]‐	PS 36:3	PS	2.48	784.5125	LIPn
PS 36:4; PS 16:0_20:4; [M‐H]‐	PS 36:4	PS	2.41	782.4968	LIPn
PS 38:3; PS 18:0_20:3; [M‐H]‐	PS 38:3	PS	2.82	812.5443	LIPn
PS 38:4; PS 18:0_20:4; [M‐H]‐	PS 38:4	PS	2.70	810.5283	LIPn
PS 38:5; PS 18:1_20:4; [M‐H]‐	PS 38:5	PS	2.43	808.5126	LIPn
PS 38:6; PS 16:0_22:6; [M‐H]‐	PS 38:6	PS	2.34	806.4974	LIPn
PS 40:4; PS 18:0_22:4; [M‐H]‐	PS 40:4	PS	2.91	838.5588	LIPn
PS 40:5 (1); PS 18:0_22:5; [M‐H]‐	PS 40:5 (1)	PS	2.70	836.5430	LIPn
PS 40:5 (2); PS 18:0_22:5; [M‐H]‐	PS 40:5 (2)	PS	2.81	836.5432	LIPn
PS 40:6; PS 18:0_22:6; [M‐H]‐	PS 40:6	PS	2.61	834.5280	LIPn
PS 40:7; [M‐H]‐	PS 40:7	PS	2.36	832.5129	LIPn
SM 32:1;2O; [M+H]+	SM 32:1;2O	SM	2.32	675.5438	LIPp
SM 32:2;2O; [M+H]+	SM 32:2;2O	SM	2.08	673.5286	LIPp
SM 33:1;2O; [M+H]+	SM 33:1;2O	SM	2.48	689.5594	LIPp
SM 34:0;2O; SM 16:0;2O/18:0; [M+H]+	SM 34:0;2O	SM	2.75	705.5905	LIPp
SM 34:1;2O; SM 18:1;2O/16:0; [M+H]+	SM 34:1;2O	SM	2.64	703.5745	LIPp
SM 34:1;3O; [M+H]+	SM 34:1;3O	SM	2.54	719.5698	LIPp
SM 34:2;2O; [M+H]+	SM 34:2;2O	SM	2.37	701.5595	LIPp
SM 34:2;3O; [M+H]+	SM 34:2;3O	SM	2.28	717.5543	LIPp
SM 36:1;2O; [M+H]+	SM 36:1;2O	SM	2.97	731.6062	LIPp
SM 36:2;2O; [M+H]+	SM 36:2;2O	SM	2.68	729.5903	LIPp
SM 36:3;2O; [M+H]+	SM 36:3;2O	SM	2.43	727.5751	LIPp
SM 38:2;2O; SM 18:2;2O/20:0; [M+H]+	SM 38:2;2O	SM	3.00	757.6216	LIPp
SM 38:4;2O; [M+H]+	SM 38:4;2O	SM	2.98	753.5880	LIPp
SM 40:2;2O; [M+H]+	SM 40:2;2O	SM	3.32	785.6532	LIPp
SM 41:2;2O; [M+H]+	SM 41:2;2O	SM	3.48	799.6693	LIPp
SM 42:1;2O; [M+H]+	SM 42:1;2O	SM	3.71	815.6997	LIPp
SM 42:2;2O; [M+H]+	SM 42:2;2O	SM	3.60	813.6843	LIPp
SM 42:2;3O; [M+H]+	SM 42:2;3O	SM	3.49	829.6799	LIPp
SM 42:3;2O; [M+H]+	SM 42:3;2O	SM	3.31	811.6688	LIPp
SM 42:3;3O; [M+H]+	SM 42:3;3O	SM	3.20	827.6643	LIPp
ST 27:1;O;S; [M‐H]‐	ST 27:1;O;S	SSulfate	1.70	465.3035	LIPn
TG 32:0; TG 8:0_12:0_12:0; [M+NH4]+	TG 32:0	TG	3.35	600.5192	LIPp‐TG
TG 34:0; TG 10:0_12:0_12:0; [M+NH4]+	TG 34:0	TG	3.67	628.5508	LIPp‐TG
TG 36:0; TG 8:0_12:0_16:0; [M+NH4]+	TG 36:0	TG	3.97	656.5821	LIPp‐TG
TG 38:0; TG 12:0_12:0_14:0; [M+NH4]+	TG 38:0	TG	4.30	684.6138	LIPp‐TG
TG 42:1; TG 8:0_16:0_18:1; [M+NH4]+	TG 42:1	TG	4.56	738.6591	LIPp‐TG
TG 42:2; TG 8:0_16:0_18:2; [M+NH4]+	TG 42:2	TG	4.33	736.6441	LIPp‐TG
TG 44:1; TG 10:0_16:0_18:1; [M+NH4]+	TG 44:1	TG	4.81	766.6917	LIPp‐TG
TG 44:2; TG 10:0_16:0_18:2; [M+NH4]+	TG 44:2	TG	4.58	764.6754	LIPp‐TG
TG 46:0; TG 14:0_16:0_16:0; [M+NH4]+	TG 46:0	TG	5.27	796.7385	LIPp‐TG
TG 46:1; TG 14:0_16:0_16:1; [M+NH4]+	TG 46:1	TG	5.05	794.7224	LIPp‐TG
TG 46:2; TG 12:0_16:1_18:1; [M+NH4]+	TG 46:2	TG	4.83	792.7069	LIPp‐TG
TG 46:3; TG 12:0_16:1_18:2; [M+NH4]+	TG 46:3	TG	4.60	790.6919	LIPp‐TG
TG 46:4; TG 10:0_18:2_18:2; [M+NH4]+	TG 46:4	TG	4.37	788.6758	LIPp‐TG
TG 48:0; TG 14:0_16:0_18:0; [M+NH4]+	TG 48:0	TG	5.48	824.7683	LIPp‐TG
TG 48:1; TG 14:0_16:0_18:1; [M+NH4]+	TG 48:1	TG	5.27	822.7532	LIPp‐TG
TG 48:2; TG 14:0_16:0_18:2; [M+NH4]+	TG 48:2	TG	5.07	820.7383	LIPp‐TG
TG 48:3; TG 14:0_16:1_18:2; [M+NH4]+	TG 48:3	TG	4.84	818.7228	LIPp‐TG
TG 48:4; TG 14:1_16:1_18:2; [M+NH4]+	TG 48:4	TG	4.63	816.7075	LIPp‐TG
TG 49:1; TG 15:0_16:0_18:1; [M+NH4]+	TG 49:1	TG	5.37	836.7692	LIPp‐TG
TG 49:2; TG 15:0_16:0_18:2; [M+NH4]+	TG 49:2	TG	5.17	834.7538	LIPp‐TG
TG 49:3; TG 15:0_16:1_18:2; [M+NH4]+	TG 49:3	TG	4.96	832.7385	LIPp‐TG
TG 50:0; TG 16:0_16:0_18:0; [M+NH4]+	TG 50:0	TG	5.67	852.8010	LIPp‐TG
TG 50:1; TG 16:0_16:0_18:1; [M+NH4]+	TG 50:1	TG	5.47	850.7850	LIPp‐TG
TG 50:2; TG 16:0_16:1_18:1; [M+NH4]+	TG 50:2	TG	5.28	848.7697	LIPp‐TG
TG 50:3; TG 16:0_16:1_18:2; [M+NH4]+	TG 50:3	TG	5.08	846.7538	LIPp‐TG
TG 50:4; TG 16:1_16:1_18:2; [M+NH4]+	TG 50:4	TG	4.87	844.7382	LIPp‐TG
TG 50:5; TG 14:0_18:2_18:3; [M+NH4]+	TG 50:5	TG	4.67	842.7230	LIPp‐TG
TG 51:1; TG 16:0_17:0_18:1; [M+NH4]+	TG 51:1	TG	5.57	864.8015	LIPp‐TG
TG 51:2; TG 16:0_17:1_18:1; [M+NH4]+	TG 51:2	TG	5.37	862.7853	LIPp‐TG
TG 51:3; TG 15:0_18:1_18:2; [M+NH4]+	TG 51:3	TG	5.18	860.7694	LIPp‐TG
TG 51:4; TG 16:1_17:1_18:2; [M+NH4]+	TG 51:4	TG	4.98	858.7542	LIPp‐TG
TG 52:1; TG 16:0_18:0_18:1; [M+NH4]+	TG 52:1	TG	5.66	878.8167	LIPp‐TG
TG 52:2; TG 16:0_18:1_18:1; [M+NH4]+	TG 52:2	TG	5.47	876.8010	LIPp‐TG
TG 52:3; TG 16:0_18:1_18:2; [M+NH4]+	TG 52:3	TG	5.29	874.7850	LIPp‐TG
TG 52:4; TG 16:1_18:1_18:2; [M+NH4]+	TG 52:4	TG	5.09	872.7697	LIPp‐TG
TG 52:5; TG 16:1_18:2_18:2; [M+NH4]+	TG 52:5	TG	4.89	870.7540	LIPp‐TG
TG 52:6; TG 16:1_18:2_18:3; [M+NH4]+	TG 52:6	TG	4.69	868.7383	LIPp‐TG
TG 53:1; TG 16:0_19:0_18:1; [M+NH4]+	TG 53:1	TG	5.74	892.8313	LIPp‐TG
TG 53:2 (1); TG 17:0_18:1_18:1; [M+NH4]+	TG 53:2 (1)	TG	5.47	890.8120	LIPp‐TG
TG 53:2 (2); TG 16:0_18:1_19:1; [M+NH4]+	TG 53:2 (2)	TG	5.56	890.8170	LIPp‐TG
TG 53:3 (1); TG 17:0_18:1_18:2; [M+NH4]+	TG 53:3 (1)	TG	5.29	888.7993	LIPp‐TG
TG 53:3 (2); TG 17:0_18:1_18:2; [M+NH4]+	TG 53:3 (2)	TG	5.38	888.8011	LIPp‐TG
TG 53:4; TG 17:1_18:1_18:2; [M+NH4]+	TG 53:4	TG	5.19	886.7861	LIPp‐TG
TG 53:5; TG 17:1_18:2_18:2; [M+NH4]+	TG 53:5	TG	5.00	884.7698	LIPp‐TG
TG 54:1; TG 16:0_20:0_18:1; [M+NH4]+	TG 54:1	TG	5.84	906.8485	LIPp‐TG
TG 54:2; TG 18:0_18:1_18:1; [M+NH4]+	TG 54:2	TG	5.66	904.8324	LIPp‐TG
TG 54:3; TG 18:0_18:1_18:2; [M+NH4]+	TG 54:3	TG	5.47	902.8165	LIPp‐TG
TG 54:4; TG 18:1_18:1_18:2; [M+NH4]+	TG 54:4	TG	5.29	900.8004	LIPp‐TG
TG 54:5 (1); TG 18:1_18:2_18:2; [M+NH4]+	TG 54:5 (1)	TG	5.10	898.7852	LIPp‐TG
TG 54:5 (2); TG 16:0_18:1_20:4; [M+NH4]+	TG 54:5 (2)	TG	5.23	898.7850	LIPp‐TG
TG 54:6 (1); TG 18:1_18:2_18:3; [M+NH4]+	TG 54:6 (1)	TG	4.91	896.7699	LIPp‐TG
TG 54:6 (2); TG 16:0_18:2_20:4; [M+NH4]+	TG 54:6 (2)	TG	5.03	896.7696	LIPp‐TG
TG 54:6 (3); TG 16:0_16:0_22:6; [M+NH4]+	TG 54:6 (3)	TG	5.13	896.7730	LIPp‐TG
TG 54:7 (1); TG 18:2_18:2_18:3; [M+NH4]+	TG 54:7 (1)	TG	4.72	894.7543	LIPp‐TG
TG 54:7 (2); TG 16:1_18:2_20:4; [M+NH4]+	TG 54:7 (2)	TG	4.81	894.7544	LIPp‐TG
TG 54:7 (3); TG 16:0_16:1_22:6; [M+NH4]+	TG 54:7 (3)	TG	4.91	894.7565	LIPp‐TG
TG 55:2; TG 18:0_18:1_19:1; [M+NH4]+	TG 55:2	TG	5.74	918.8486	LIPp‐TG
TG 55:3; TG 19:0_18:1_18:2; [M+NH4]+	TG 55:3	TG	5.57	916.8325	LIPp‐TG
TG 55:4; TG 18:1_19:1_18:2; [M+NH4]+	TG 55:4	TG	5.39	914.8180	LIPp‐TG
TG 55:5; TG 18:1_18:2_19:2; [M+NH4]+	TG 55:5	TG	5.22	912.8011	LIPp‐TG
TG 56:2; TG 18:0_18:1_20:1; [M+NH4]+	TG 56:2	TG	5.83	932.8643	LIPp‐TG
TG 56:3; TG 18:1_18:1_20:1; [M+NH4]+	TG 56:3	TG	5.65	930.8480	LIPp‐TG
TG 56:4; TG 18:1_20:1_18:2; [M+NH4]+	TG 56:4	TG	5.48	928.8320	LIPp‐TG
TG 56:5 (1); TG 18:1_18:2_20:2; [M+NH4]+	TG 56:5 (1)	TG	5.31	926.8160	LIPp‐TG
TG 56:5 (2); TG 18:0_18:1_20:4; [M+NH4]+	TG 56:5 (2)	TG	5.44	926.8189	LIPp‐TG
TG 56:6; TG 18:1_18:1_20:4; [M+NH4]+	TG 56:6	TG	5.23	924.8002	LIPp‐TG
TG 56:7 (1); TG 18:1_18:2_20:4; [M+NH4]+	TG 56:7 (1)	TG	5.04	922.7847	LIPp‐TG
TG 56:7 (2); TG 16:0_18:1_22:6; [M+NH4]+	TG 56:7 (2)	TG	5.12	922.7866	LIPp‐TG
TG 56:8 (1); TG 18:2_18:2_20:4; [M+NH4]+	TG 56:8 (1)	TG	4.85	920.7689	LIPp‐TG
TG 56:8 (2); TG 16:0_18:2_22:6; [M+NH4]+	TG 56:8 (2)	TG	4.93	920.7734	LIPp‐TG
TG 58:3; TG 22:0_18:1_18:2; [M+NH4]+	TG 58:3	TG	5.82	958.8793	LIPp‐TG
TG 58:4; TG 18:1_22:1_18:2; [M+NH4]+	TG 58:4	TG	5.66	956.8629	LIPp‐TG
TG 58:7; TG 18:1_18:2_22:4; [M+NH4]+	TG 58:7	TG	5.20	950.8160	LIPp‐TG
TG 58:8 (1); TG 18:1_18:2_22:5; [M+NH4]+	TG 58:8 (1)	TG	5.03	948.8005	LIPp‐TG
TG 58:8 (2); TG 18:1_18:1_22:6; [M+NH4]+	TG 58:8 (2)	TG	5.12	948.8021	LIPp‐TG
TG 58:9; TG 18:1_18:2_22:6; [M+NH4]+	TG 58:9	TG	4.94	946.7853	LIPp‐TG
TG 58:10; TG 18:2_18:2_22:6; [M+NH4]+	TG 58:10	TG	4.74	944.7679	LIPp‐TG

Abbreviations: CAR, acylcarnitine; Cer‐NS, ceramide non‐hydroxy fatty acid‐sphingosine; Chol, cholesterol; DG, diacylglycerol; EtherLPE, ether‐linked lysophosphatidylethanolamine; EtherPC, ether‐linked phosphatidylcholine; EtherPE, ether‐linked phosphatidylethanolamine; FA, free fatty acid; HSL, hormone‐sensitive lipase; LPC, lysophophatidylcholine; LPE, lysophosphatidylethanolamine; MG, monoacylglycerol; NAE, N‐acyl ethanolamine; OxPC, oxidized phosphatidylcholine; OxPE, oxidized phosphatidylethanolamine; PC, phosphatidylcholine; PE, phosphatidylethanolamine; PEtOH, phosphatidylethanol; PG, phosphatidylglycerol; PI, phosphatidylinositol; PS, phosphatidylserine; SM, sphingomyelin; SSulfate, sterol sulfate; TG, triacylglycerols.

**TABLE A3:** List of annotated fatty acid esters of hydroxy fatty acids in plasma samples.

Metabolite name (full)	Metabolite name	Metabolite name (regioisomer annotation)	Sublass	Average retention time (min)	Average *m/z*	Platform
FAHFA 32:0;O (1); FAHFA 16:0/16:0;O; [M‐H]‐	FAHFA 32:0;O (1)	13/12‐PAHPA	FAHFA	16.06	509.4587	FAHFA
FAHFA 32:0;O (2); FAHFA 16:0/16:0;O; [M‐H]‐	FAHFA 32:0;O (2)	9‐PAHPA	FAHFA	16.95	509.4590	FAHFA
FAHFA 32:1;O (1); FAHFA 16:1/16:0;O; [M‐H]‐	FAHFA 32:1;O (1)	9‐POHPA	FAHFA	13.41	507.4432	FAHFA
FAHFA 32:1;O (2); FAHFA 16:1/16:0;O; [M‐H]‐	FAHFA 32:1;O (2)	8‐POHPA	FAHFA	13.78	507.4431	FAHFA
FAHFA 32:1;O (3); FAHFA 16:0/16:1;O; [M‐H]‐	FAHFA 32:1;O (3)	13/12‐PAHPO	FAHFA	14.15	507.4430	FAHFA
FAHFA 32:2;O (2); FAHFA 16:1/16:1;O; [M‐H]‐	FAHFA 32:2;O (2)	10‐POHPO	FAHFA	11.97	505.4273	FAHFA
FAHFA 32:2;O (3); FAHFA 16:1/16:1;O; [M‐H]‐	FAHFA 32:2;O (3)	9‐POHPO	FAHFA	12.28	505.4273	FAHFA
FAHFA 34:0;O (1); FAHFA 16:0/18:0;O; [M‐H]‐	FAHFA 34:0;O (1)	13/12‐PAHSA	FAHFA	19.23	537.4905	FAHFA
FAHFA 34:0;O (2); FAHFA 18:0/16:0;O; [M‐H]‐	FAHFA 34:0;O (2)	13/12‐SAHPA	FAHFA	19.90	537.4912	FAHFA
FAHFA 34:0;O (3); FAHFA 16:0/18:0;O; [M‐H]‐	FAHFA 34:0;O (3)	10‐PAHSA	FAHFA	20.52	537.4904	FAHFA
FAHFA 34:0;O (4); FAHFA 16:0/18:0;O; [M‐H]‐	FAHFA 34:0;O (4)	9‐PAHSA	FAHFA	21.07	537.4913	FAHFA
FAHFA 34:1;O (1); FAHFA 18:1/16:0;O; [M‐H]‐	FAHFA 34:1;O (1)	13/12‐OAHPA	FAHFA	15.71	535.4745	FAHFA
FAHFA 34:1;O (2); FAHFA 16:0/18:1;O; [M‐H]‐	FAHFA 34:1;O (2)	12‐PAHOA	FAHFA	15.97	535.4739	FAHFA
FAHFA 34:1;O (4); FAHFA 16:0/18:1;O; [M‐H]‐	FAHFA 34:1;O (4)	11‐PAHOA	FAHFA	17.08	535.4746	FAHFA
FAHFA 34:1;O (5); FAHFA 16:0/18:1;O; [M‐H]‐	FAHFA 34:1;O (5)	9‐PAHOA	FAHFA	17.31	535.4745	FAHFA
FAHFA 34:2;O (1); FAHFA 18:2/16:0;O; [M‐H]‐	FAHFA 34:2;O (1)	13/12‐LAHPA	FAHFA	13.31	533.4588	FAHFA
FAHFA 34:2;O (3); FAHFA 16:0/18:2;O; [M‐H]‐	FAHFA 34:2;O (3)	13‐PAHLA	FAHFA	15.30	533.4589	FAHFA
FAHFA 34:2;O (4); FAHFA 16:0/18:2;O; [M‐H]‐	FAHFA 34:2;O (4)	9‐PAHLA	FAHFA	15.79	533.4586	FAHFA
FAHFA 34:3;O (2); FAHFA 16:1/18:2;O; [M‐H]‐	FAHFA 34:3;O (2)	13‐POHLA	FAHFA	12.61	531.4426	FAHFA
FAHFA 36:0;O; FAHFA 18:0/18:0;O; [M‐H]‐	FAHFA 36:0;O	9‐SAHSA	FAHFA	25.61	565.5214	FAHFA
FAHFA 36:1;O (1); FAHFA 18:1/18:0;O; [M‐H]‐	FAHFA 36:1;O (1)	13/12‐OAHSA	FAHFA	18.95	563.5059	FAHFA
FAHFA 36:1;O (2); FAHFA 18:1/18:0;O; [M‐H]‐	FAHFA 36:1;O (2)	11‐OAHSA	FAHFA	19.56	563.5064	FAHFA
FAHFA 36:1;O (3); FAHFA 18:1/18:0;O; [M‐H]‐	FAHFA 36:1;O (3)	10‐OAHSA	FAHFA	20.00	563.5063	FAHFA
FAHFA 36:1;O (4); FAHFA 18:0/18:1;O; [M‐H]‐	FAHFA 36:1;O (4)	12‐SAHOA	FAHFA	20.55	563.5063	FAHFA
FAHFA 36:2;O (1); FAHFA 18:1/18:1;O; [M‐H]‐	FAHFA 36:2;O (1)	12‐OAHOA	FAHFA	16.28	561.4904	FAHFA
FAHFA 36:2;O (2); FAHFA 18:1/18:1;O; [M‐H]‐	FAHFA 36:2;O (2)	11/10‐OAHOA	FAHFA	16.90	561.4907	FAHFA
FAHFA 36:2;O (3); FAHFA 18:1/18:1;O; [M‐H]‐	FAHFA 36:2;O (3)	9‐OAHOA	FAHFA	17.62	561.4899	FAHFA
FAHFA 36:3;O (1); FAHFA 18:2/18:1;O; [M‐H]‐	FAHFA 36:3;O (1)	12‐LAHOA	FAHFA	13.74	559.4746	FAHFA
FAHFA 36:3;O (2); FAHFA 18:2/18:1;O; [M‐H]‐	FAHFA 36:3;O (2)	11/10‐LAHOA	FAHFA	14.19	559.4744	FAHFA
FAHFA 36:4;O; FAHFA 18:2/18:2;O; [M‐H]‐	FAHFA 36:4;O	13‐LAHLA	FAHFA	12.72	557.4589	FAHFA
FAHFA 38:5;O; FAHFA 20:4/18:1;O; [M‐H]‐	FAHFA 38:5;O	11/10‐AAHOA	FAHFA	12.48	583.4744	FAHFA
FAHFA 40:7;O; FAHFA 22:6/18:1;O; [M‐H]‐	FAHFA 40:7;O	11/10‐DHAHOA	FAHFA	11.50	607.4746	FAHFA

Abbreviations: AAHOA, arachidonic acid esters of hydroxy oleic acids; DHAHOA, docosahexaenoic acid esters of hydroxy oleic acids; FAHFA, fatty acid esters of hydroxy fatty acids; LAHLA, linoleic acid esters of hydroxy linoleic acids; LAHOA, linoleic acid esters of hydroxy oleic acids; LAHPA, linoleic acid esters of hydroxy palmitic acids; OAHOA, oleic acid esters of hydroxy oleic acids; OAHPA, oleic acid esters of hydroxy palmitic acids; OAHSA, oleic acid esters of hydroxy stearic acids; PAHLA, palmitic acid esters of hydroxy linoleic acids; PAHOA, palmitic acid esters of hydroxy oleic acids; PAHPA, palmitic acid esters of hydroxy palmitic acids; PAHPO, palmitic acid esters of hydroxy palmitoleic acids; PAHSA, palmitic acid esters of hydroxy stearic acids; POHLA, palmitoleic acid esters of hydroxy linoleic acids; POHPA, palmitoleic acid esters of hydroxy palmitic acids; POHPO, palmitoleic acid esters of hydroxy palmitoleic acids; SAHOA, stearic acid esters of hydroxy oleic acids; SAHPA, stearic acid esters of hydroxy palmitic acids; SAHSA, stearic acid esters of hydroxy stearic acids.

**TABLE A4:** List of annotated fatty acid esters of hydroxy fatty acids in epididymal adipose samples.

Metabolite name (full)	Metabolite name	Metabolite name (regioisomer annotation)	Sublass	Average retention time (min)	Average *m/z*	Platform
FAHFA 32:0;O (1); FAHFA 16:0/16:0;O; [M‐H]‐	FAHFA 32:0;O (1)	13/12‐PAHPA	FAHFA	15.13	509.4583	FAHFA
FAHFA 32:0;O (3); FAHFA 16:0/16:0;O; [M‐H]‐	FAHFA 32:0;O (3)	9‐PAHPA	FAHFA	15.94	509.4580	FAHFA
FAHFA 32:0;O (4); FAHFA 16:0/16:0;O; [M‐H]‐	FAHFA 32:0;O (4)	8‐PAHPA	FAHFA	16.31	509.4583	FAHFA
FAHFA 32:0;O (5); FAHFA 16:0/16:0;O; [M‐H]‐	FAHFA 32:0;O (5)	7‐PAHPA	FAHFA	16.66	509.4583	FAHFA
FAHFA 32:1;O (1); FAHFA 16:1/16:0;O; [M‐H]‐	FAHFA 32:1;O (1)	13/12‐POHPA	FAHFA	12.49	507.4427	FAHFA
FAHFA 32:1;O (5); FAHFA 16:0/16:1;O; [M‐H]‐	FAHFA 32:1;O (5)	12‐PAHPO	FAHFA	13.65	507.4422	FAHFA
FAHFA 32:1;O (6); FAHFA 16:0/16:1;O; [M‐H]‐	FAHFA 32:1;O (6)	11‐PAHPO	FAHFA	13.92	507.4424	FAHFA
FAHFA 32:1;O (7); FAHFA 16:0/16:1;O; [M‐H]‐	FAHFA 32:1;O (7)	10‐PAHPO	FAHFA	14.19	507.4428	FAHFA
FAHFA 32:1;O (8); FAHFA 16:0/16:1;O; [M‐H]‐	FAHFA 32:1;O (8)	9‐PAHPO	FAHFA	14.77	507.4424	FAHFA
FAHFA 32:2;O (2); FAHFA 16:1/16:1;O; [M‐H]‐	FAHFA 32:2;O (2)	12‐POHPO	FAHFA	11.28	505.4270	FAHFA
FAHFA 32:2;O (3); FAHFA 16:1/16:1;O; [M‐H]‐	FAHFA 32:2;O (3)	10‐POHPO	FAHFA	11.54	505.4273	FAHFA
FAHFA 32:2;O (4); FAHFA 16:1/16:1;O; [M‐H]‐	FAHFA 32:2;O (4)	9‐POHPO	FAHFA	11.85	505.4271	FAHFA
FAHFA 34:0;O (1); FAHFA 16:0/18:0;O; [M‐H]‐	FAHFA 34:0;O (1)	13/12‐PAHSA	FAHFA	18.11	537.4895	FAHFA
FAHFA 34:0;O (2); FAHFA 16:0/18:0;O; [M‐H]‐	FAHFA 34:0;O (2)	11‐PAHSA	FAHFA	18.67	537.4895	FAHFA
FAHFA 34:0;O (3); FAHFA 16:0/18:0;O; [M‐H]‐	FAHFA 34:0;O (3)	10‐PAHSA	FAHFA	19.08	537.4896	FAHFA
FAHFA 34:0;O (4); FAHFA 16:0/18:0;O; [M‐H]‐	FAHFA 34:0;O (4)	9‐PAHSA	FAHFA	19.54	537.4898	FAHFA
FAHFA 34:0;O (5); FAHFA 16:0/18:0;O; [M‐H]‐	FAHFA 34:0;O (5)	8‐PAHSA	FAHFA	20.01	537.4893	FAHFA
FAHFA 34:1;O (1); FAHFA 18:1/16:0;O; [M‐H]‐	FAHFA 34:1;O (1)	13/12‐OAHPA	FAHFA	14.81	535.4737	FAHFA
FAHFA 34:1;O (2); FAHFA 18:1/16:0;O; [M‐H]‐	FAHFA 34:1;O (2)	9‐OAHPA	FAHFA	15.02	535.4738	FAHFA
FAHFA 34:1;O (4); FAHFA 16:0/18:1;O; [M‐H]‐	FAHFA 34:1;O (4)	12‐PAHOA	FAHFA	15.60	535.4742	FAHFA
FAHFA 34:1;O (5); FAHFA 16:0/18:1;O; [M‐H]‐	FAHFA 34:1;O (5)	11/10‐PAHOA	FAHFA	16.23	535.4739	FAHFA
FAHFA 34:1;O (6); FAHFA 16:0/18:1;O; [M‐H]‐	FAHFA 34:1;O (6)	9‐PAHOA	FAHFA	16.70	535.4740	FAHFA
FAHFA 34:2;O (1); FAHFA 18:2/16:0;O; [M‐H]‐	FAHFA 34:2;O (1)	13/12‐LAHPA	FAHFA	12.58	533.4582	FAHFA
FAHFA 34:2;O (2); FAHFA 16:1/18:1;O; [M‐H]‐	FAHFA 34:2;O (2)	12‐POHOA	FAHFA	12.84	533.4583	FAHFA
FAHFA 34:2;O (4); FAHFA 16:1/18:1;O; [M‐H]‐	FAHFA 34:2;O (4)	11/10‐POHOA	FAHFA	13.26	533.4580	FAHFA
FAHFA 34:2;O (7); FAHFA 16:0/18:2;O; [M‐H]‐	FAHFA 34:2;O (7)	13‐PAHLA	FAHFA	14.46	533.4583	FAHFA
FAHFA 34:2;O (8); FAHFA 16:0/18:2;O; [M‐H]‐	FAHFA 34:2;O (8)	9‐PAHLA	FAHFA	14.92	533.4583	FAHFA
FAHFA 34:3;O (2); FAHFA 18:2/16:1;O; [M‐H]‐	FAHFA 34:3;O (2)	12‐LAHPO	FAHFA	11.34	531.4428	FAHFA
FAHFA 34:3;O (4); FAHFA 16:1/18:2;O; [M‐H]‐	FAHFA 34:3;O (4)	13‐POHLA	FAHFA	12.05	531.4424	FAHFA
FAHFA 34:3;O (6); FAHFA 18:1/16:2;O; [M‐H]‐	FAHFA 34:3;O (6)	9‐LAHPO	FAHFA	12.65	531.4424	FAHFA
FAHFA 36:0;O (2); FAHFA 18:0/18:0;O; [M‐H]‐	FAHFA 36:0;O (2)	10‐SAHSA	FAHFA	23.96	565.5208	FAHFA
FAHFA 36:0;O (3); FAHFA 18:0/18:0;O; [M‐H]‐	FAHFA 36:0;O (3)	9‐SAHSA	FAHFA	24.57	565.5210	FAHFA
FAHFA 36:0;O (4); FAHFA 18:0/18:0;O; [M‐H]‐	FAHFA 36:0;O (4)	8‐SAHSA	FAHFA	25.16	565.5209	FAHFA
FAHFA 36:1;O (1); FAHFA 18:1/18:0;O; [M‐H]‐	FAHFA 36:1;O (1)	13/12‐OAHSA	FAHFA	17.67	563.5050	FAHFA
FAHFA 36:1;O (2); FAHFA 18:1/18:0;O; [M‐H]‐	FAHFA 36:1;O (2)	11‐OAHSA	FAHFA	18.22	563.5051	FAHFA
FAHFA 36:1;O (3); FAHFA 18:1/18:0;O; [M‐H]‐	FAHFA 36:1;O (3)	10‐OAHSA	FAHFA	18.59	563.5051	FAHFA
FAHFA 36:1;O (4); FAHFA 18:1/18:0;O; [M‐H]‐	FAHFA 36:1;O (4)	9‐OAHSA	FAHFA	19.08	563.5050	FAHFA
FAHFA 36:1;O (5); FAHFA 18:0/18:1;O; [M‐H]‐	FAHFA 36:1;O (5)	11/10‐SAHOA	FAHFA	19.96	563.5049	FAHFA
FAHFA 36:2;O (1); FAHFA 18:2/18:0;O; [M‐H]‐	FAHFA 36:2;O (1)	13/12‐LAHSA	FAHFA	14.71	561.4895	FAHFA
FAHFA 36:2;O (2); FAHFA 18:1/18:1;O; [M‐H]‐	FAHFA 36:2;O (2)	12‐OAHOA	FAHFA	15.28	561.4897	FAHFA
FAHFA 36:2;O (3); FAHFA 18:1/18:1;O; [M‐H]‐	FAHFA 36:2;O (3)	11/10‐OAHOA	FAHFA	15.89	561.4892	FAHFA
FAHFA 36:2;O (4); FAHFA 18:1/18:1;O; [M‐H]‐	FAHFA 36:2;O (4)	9‐OAHOA	FAHFA	16.41	561.4893	FAHFA
FAHFA 36:2;O (5); FAHFA 18:0/18:2;O; [M‐H]‐	FAHFA 36:2;O (5)	13‐SAHLA	FAHFA	17.58	561.4894	FAHFA
FAHFA 36:3;O (1); FAHFA 18:2/18:1;O; [M‐H]‐	FAHFA 36:3;O (1)	12‐LAHOA	FAHFA	12.94	559.4743	FAHFA
FAHFA 36:3;O (2); FAHFA 18:2/18:1;O; [M‐H]‐	FAHFA 36:3;O (2)	11/10‐LAHOA	FAHFA	13.40	559.4737	FAHFA
FAHFA 36:3;O (3); FAHFA 18:2/18:1;O; [M‐H]‐	FAHFA 36:3;O (3)	9‐LAHOA	FAHFA	13.78	559.4737	FAHFA
FAHFA 36:3;O (4); FAHFA 18:1/18:2;O; [M‐H]‐	FAHFA 36:3;O (4)	13‐OAHLA	FAHFA	14.16	559.4736	FAHFA
FAHFA 36:3;O (5); FAHFA 18:1/18:2;O; [M‐H]‐	FAHFA 36:3;O (5)	9‐OAHLA	FAHFA	14.64	559.4739	FAHFA
FAHFA 36:4;O (4); FAHFA 18:2/18:2;O; [M‐H]‐	FAHFA 36:4;O (4)	13‐LAHLA	FAHFA	12.17	557.4584	FAHFA
FAHFA 38:4;O (3); FAHFA 20:4/18:0;O; [M‐H]‐	FAHFA 38:4;O (3)	10‐AAHSA	FAHFA	14.29	585.4891	FAHFA
FAHFA 38:5;O (2); FAHFA 20:4/18:1;O; [M‐H]‐	FAHFA 38:5;O (2)	12‐AAHOA	FAHFA	11.95	583.4742	FAHFA
FAHFA 38:5;O (3); FAHFA 20:4/18:1;O; [M‐H]‐	FAHFA 38:5;O (3)	11/10‐AAHOA	FAHFA	12.31	583.4737	FAHFA
FAHFA 38:6;O (3); FAHFA 20:4/18:2;O; [M‐H]‐	FAHFA 38:6;O (3)	13‐AAHLA	FAHFA	11.31	581.4581	FAHFA
FAHFA 40:8;O; FAHFA 22:6/18:2;O; [M‐H]‐	FAHFA 40:8;O	13‐DHAHLA	FAHFA	10.55	605.4585	FAHFA

Abbreviations: AAHLA, arachidonic acid esters of hydroxy linoleic acids; AAHOA, arachidonic acid esters of hydroxy oleic acids; AAHSA, arachidonic acid esters of hydroxy stearic acids; DHAHLA, docosahexaenoic acid esters of hydroxy linoleic acids; FAHFA, fatty acid esters of hydroxy fatty acids; LAHLA, linoleic acid esters of hydroxy linoleic acids; LAHOA, linoleic acid esters of hydroxy oleic acids; LAHPA, linoleic acid esters of hydroxy palmitic acids; LAHPO, linoleic acid esters of hydroxy palmitoleic acids; LAHSA, linoleic acid esters of hydroxy stearic acids; OAHLA, oleic acid esters of hydroxy linoleic acids; OAHOA, oleic acid esters of hydroxy oleic acids; OAHPA, oleic acid esters of hydroxy palmitic acids; OAHSA, oleic acid esters of hydroxy stearic acids; PAHLA, palmitic acid esters of hydroxy linoleic acids; PAHOA, palmitic acid esters of hydroxy oleic acids; PAHPA, palmitic acid esters of hydroxy palmitic acids; PAHPO, palmitic acid esters of hydroxy palmitoleic acids; PAHSA, palmitic acid esters of hydroxy stearic acids; POHLA, palmitoleic acid esters of hydroxy linoleic acids; POHOA, palmitoleic acid esters of hydroxy oleic acids; POHPA, palmitoleic acid esters of hydroxy palmitic acids; POHPO, palmitoleic acid esters of hydroxy palmitoleic acids; SAHLA, stearic acid esters of hydroxy linoleic acids; SAHOA, stearic acid esters of hydroxy oleic acids; SAHSA, stearic acid esters of hydroxy stearic acids.

## References

[eph70375-bib-0001] Arner, P. , Liljeqvist, L. , & Östman, J. (1976). Metabolism of mono‐ and diacylglycerols in subcutaneous adipose tissue of obese and normal‐weight subjects. Acta Medica Scandinavica, 200(3), 187–194. 10.1111/j.0954-6820.1976.tb08218.x 184690

[eph70375-bib-0002] Arner, P. , Pettersson, A. , Mitchell, P. J. , Dunbar, J. D. , Kharitonenkov, A. , & Rydén, M. (2008). FGF21 attenuates lipolysis in human adipocytes—a possible link to improved insulin sensitivity. FEBS Letters, 582(12), 1725–1730. 10.1016/j.febslet.2008.04.038 18460341

[eph70375-bib-0003] Aryal, P. , Syed, I. , Lee, J. , Patel, R. , Nelson, A. T. , Siegel, D. , Saghatelian, A. , & Kahn, B. B. (2021). Distinct biological activities of isomers from several families of branched fatty acid esters of hydroxy fatty acids (FAHFAs). Journal of Lipid Research, 62, 100108. 10.1016/j.jlr.2021.100108 PMC8479484 34418413

[eph70375-bib-0004] Benz, V. , Bloch, M. , Wardat, S. , Böhm, C. , Maurer, L. , Mahmoodzadeh, S. , Wiedmer, P. , Spranger, J. , Foryst‐Ludwig, A. , & Kintscher, U. (2012). Sexual dimorphic regulation of body weight dynamics and adipose tissue lipolysis. PLoS ONE, 7(5), e37794. 10.1371/journal.pone.0037794 PMC3360591 22662224

[eph70375-bib-0005] Brandt, N. , Dethlefsen, M. M. , Bangsbo, J. , & Pilegaard, H. (2017). PGC‐1alpha and exercise intensity dependent adaptations in mouse skeletal muscle. PLoS ONE, 12(10), e0185993. 10.1371/journal.pone.0185993 PMC5648136 29049322

[eph70375-bib-0006] Brejchova, K. , Radner, F. P. W. , Balas, L. , Paluchova, V. , Cajka, T. , Chodounska, H. , Kudova, E. , Schratter, M. , Schreiber, R. , Durand, T. , Zechner, R. , & Kuda, O. (2021). Distinct roles of adipose triglyceride lipase and hormone‐sensitive lipase in the catabolism of triacylglycerol estolides. Proceedings of the National Academy of Science of the United States of America, 118(2), e2020999118. 10.1073/pnas.2020999118 PMC7812821 33372146

[eph70375-bib-0007] Brezinova, M. , Cajka, T. , Oseeva, M. , Stepan, M. , Dadova, K. , Rossmeislova, L. , Matous, M. , Siklova, M. , Rossmeisl, M. , & Kuda, O. (2020). Exercise training induces insulin‐sensitizing PAHSAs in adipose tissue of elderly women. Biochimica et Biophysica Acta (BBA) ‐ Molecular and Cell Biology of Lipids, 1865(2), 158576. 10.1016/j.bbalip.2019.158576 31740387

[eph70375-bib-0008] Brooks, G. A (1997). Importance of the ‘crossover’ concept in exercise metabolism. Clinical and Experimental Pharmacology & Physiology, 24(11), 889–895. 10.1111/j.1440-1681.1997.tb02712.x 9363377

[eph70375-bib-0009] Brooks, G. A. , & Mercier, J. (1994). Balance of carbohydrate and lipid utilization during exercise: The “crossover” concept. Journal of Applied Physiology, 76(6), 2253–2261. 10.1152/jappl.1994.76.6.2253 7928844

[eph70375-bib-0010] Choi, S. W. , Oh, H. , Park, S. Y. , Cho, W. , Abd El‐Aty, A. M. , Hacimuftuoglu, A. , Jeong, J. H. , & Jung, T. W. (2023). Myokine musclin alleviates lipid accumulation in 3T3‐L1 adipocytes through PKA/p38‐mediated upregulation of lipolysis and suppression of lipogenesis. Biochemical and Biophysical Research Communications, 642, 113–117. 10.1016/j.bbrc.2022.12.056 36566562

[eph70375-bib-0011] Chong, M. F. , Hodson, L. , Bickerton, A. S. , Roberts, R. , Neville, M. , Karpe, F. , Frayn, K. N. , & Fielding, B. A. (2008). Parallel activation of de novo lipogenesis and stearoyl‐CoA desaturase activity after 3 d of high‐carbohydrate feeding. The American Journal of Clinical Nutrition, 87(4), 817–823. 10.1093/ajcn/87.4.817 18400702

[eph70375-bib-0012] Chow, L. S. , Gerszten, R. E. , Taylor, J. M. , Pedersen, B. K. , van Praag, H. , Trappe, S. , Febbraio, M. A. , Galis, Z. S. , Gao, Y. , Haus, J. M. , Lanza, I. R. , Lavie, C. J. , Lee, C. H. , Lucia, A. , Moro, C. , Pandey, A. , Robbins, J. M. , Stanford, K. I. , Thackray, A. E. , … Snyder, M. P. (2022). Exerkines in health, resilience and disease. Nature Reviews Endocrinology, 18(5), 273–289. 10.1038/s41574-022-00641-2 PMC9554896 35304603

[eph70375-bib-0013] Collins, S. , Daniel, K. W. , Petro, A. E. , & Surwit, R. S. (1997). Strain‐specific response to beta3‐adrenergic receptor agonist treatment of diet‐induced obesity in mice. Endocrinology, 138(1), 405–413. 10.1210/endo.138.1.4829 8977430

[eph70375-bib-0014] Crandall, D. L. , Goldstein, B. M. , Huggins, F. , & Cervoni, P. (1984). Adipocyte blood flow: Influence of age, anatomic location, and dietary manipulation. American Journal of Physiology, 247(1), R46–R51. 10.1152/ajpregu.1984.247.1.R46 6742235

[eph70375-bib-0015] Dong, P. , Zhang, Y. , Liu, C. X. , Yan, D. Y. , Xu, X. , Li, A. H. , & Jin, L. M. (2025). Association between human milk fatty acid esters of hydroxy fatty acids and maternal body mass index and early infant growth: A longitudinal study. Journal of Nutrition, 155(7), 2290–2299. 10.1016/j.tjnut.2025.05.022 40412759

[eph70375-bib-0016] Erikci Ertunc, M. , Kok, B. P. , Parsons, W. H. , Wang, J. G. , Tan, D. , Donaldson, C. J. , Pinto, A. F. M. , Vaughan, J. M. , Ngo, N. , Lum, K. M. , Henry, C. L. , Coppola, A. R. , Niphakis, M. J. , Cravatt, B. F. , Saez, E. , & Saghatelian, A. (2020). AIG1 and ADTRP are endogenous hydrolases of fatty acid esters of hydroxy fatty acids (FAHFAs) in mice. Journal of Biological Chemistry, 295(19), 5891–5905. 10.1074/jbc.RA119.012145 PMC7196635 32152231

[eph70375-bib-0017] Ertunc, M. E. , Konduri, S. , Ma, Z. , Pinto, A. F. M. , Donaldson, C. J. , Momper, J. , Siegel, D. , & Saghatelian, A. (2024). Acute inflammation upregulates FAHFAs in adipose tissue and in differentiated adipocytes. Journal of Biological Chemistry, 300(12), 107972. 10.1016/j.jbc.2024.107972 PMC11647607 39510180

[eph70375-bib-0018] Flachs, P. , Rühl, R. , Hensler, M. , Janovska, P. , Zouhar, P. , Kus, V. , Macek Jilkova, Z. , Papp, E. , Kuda, O. , Svobodova, M. , Rossmeisl, M. , Tsenov, G. , Mohamed‐Ali, V. , & Kopecky, J. (2011). Synergistic induction of lipid catabolism and anti‐inflammatory lipids in white fat of dietary obese mice in response to calorie restriction and n‐3 fatty acids. Diabetologia, 54(11), 2626–2638. 10.1007/s00125-011-2233-2 21779874

[eph70375-bib-0019] Fuller, S. E. , Huang, T. Y. , Simon, J. , Batdorf, H. M. , Essajee, N. M. , Scott, M. C. , Waskom, C. M. , Brown, J. M. , Burke, S. J. , Collier, J. J. , & Noland, R. C. (2019). Low‐intensity exercise induces acute shifts in liver and skeletal muscle substrate metabolism but not chronic adaptations in tissue oxidative capacity. Journal of Applied Physiology, 127(1), 143–156. 10.1152/japplphysiol.00820.2018 PMC6692746 31095457

[eph70375-bib-0020] Furukawa, S. , Fujita, T. , Shimabukuro, M. , Iwaki, M. , Yamada, Y. , Nakajima, Y. , Nakayama, O. , Makishima, M. , Matsuda, M. , & Shimomura, I. (2004). Increased oxidative stress in obesity and its impact on metabolic syndrome. The Journal of Clinical Investigation, 114(12), 1752–1761. 10.1172/JCI21625 PMC535065 15599400

[eph70375-bib-0021] Hammarstedt, A. , Syed, I. , Vijayakumar, A. , Eliasson, B. , Gogg, S. , Kahn, B. B. , & Smith, U. (2018). Adipose tissue dysfunction is associated with low levels of the novel palmitic acid hydroxystearic acids. Scientific Reports, 8(1), 15757. 10.1038/s41598-018-34113-3 PMC6202399 30361530

[eph70375-bib-0022] Horakova, O. , Medrikova, D. , van Schothorst, E. M. , Bunschoten, A. , Flachs, P. , Kus, V. , Kuda, O. , Bardova, K. , Janovska, P. , Hensler, M. , Rossmeisl, M. , Wang‐Sattler, R. , Prehn, C. , Adamski, J. , Illig, T. , Keijer, J. , & Kopecky, J. (2012). Preservation of metabolic flexibility in skeletal muscle by a combined use of n‐3 PUFA and rosiglitazone in dietary obese mice. PLoS ONE, 7(8), e43764. 10.1371/journal.pone.0043764 PMC3432031 22952760

[eph70375-bib-0023] Horakova, O. , Sistilli, G. , Kalendova, V. , Bardova, K. , Mitrovic, M. , Cajka, T. , Irodenko, I. , Janovska, P. , Lackner, K. , Kopecky, J. , & Rossmeisl, M. (2023). Thermoneutral housing promotes hepatic steatosis in standard diet‐fed C57BL/6N mice, with a less pronounced effect on NAFLD progression upon high‐fat feeding. Frontiers in Endocrinology, 14, 1205703. 10.3389/fendo.2023.1205703 PMC10369058 37501785

[eph70375-bib-0024] Hudgins, L. C. , Hellerstein, M. , Seidman, C. , Neese, R. , Diakun, J. , & Hirsch, J. (1996). Human fatty acid synthesis is stimulated by a eucaloric low fat, high carbohydrate diet. Journal of Clinical Investigation, 97(9), 2081–2091. 10.1172/JCI118645 PMC507283 8621798

[eph70375-bib-0025] Janovska, P. , Melenovsky, V. , Svobodova, M. , Havlenova, T. , Kratochvilova, H. , Haluzik, M. , Hoskova, E. , Pelikanova, T. , Kautzner, J. , Monzo, L. , Jurcova, I. , Adamcova, K. , Lenkova, L. , Buresova, J. , Rossmeisl, M. , Kuda, O. , Cajka, T. , & Kopecky, J. (2020). Dysregulation of epicardial adipose tissue in cachexia due to heart failure: The role of natriuretic peptides and cardiolipin. Journal of Cachexia, Sarcopenia and Muscle, 11(6), 1614–1627. 10.1002/jcsm.12631 PMC7749591 33084249

[eph70375-bib-0026] Ji, L. L. , Gomez‐Cabrera, M. C. , & Vina, J. (2006). Exercise and hormesis: Activation of cellular antioxidant signaling pathway. Annals of the New York Academy of Sciences, 1067(1), 425–435. 10.1196/annals.1354.061 16804022

[eph70375-bib-0027] Kellerer, T. , Kleigrewe, K. , Brandl, B. , Hofmann, T. , Hauner, H. , & Skurk, T. (2021). Fatty acid esters of hydroxy fatty acids (FAHFAs) are associated with Diet, BMI, and age. Frontiers in Nutrition, 8, 691401. 10.3389/fnut.2021.691401 PMC8310931 34322511

[eph70375-bib-0028] Kovacova, Z. , Tharp, W. G. , Liu, D. , Wei, W. , Xie, H. , Collins, S. , & Pratley, R. E. (2016). Adipose tissue natriuretic peptide receptor expression is related to insulin sensitivity in obesity and diabetes. Obesity (Silver Spring), 24(4), 820–828. 10.1002/oby.21418 PMC5067565 26887289

[eph70375-bib-0029] Kuda, O. , Brezinova, M. , Silhavy, J. , Landa, V. , Zidek, V. , Dodia, C. , Kreuchwig, F. , Vrbacky, M. , Balas, L. , Durand, T. , Hübner, N. , Fisher, A. B. , Kopecky, J. , & Pravenec, M. (2018). Nrf2‐mediated antioxidant defense and peroxiredoxin 6 are linked to biosynthesis of palmitic acid ester of 9‐hydroxystearic acid. Diabetes, 67(6), 1190–1199. 10.2337/db17-1087 PMC6463562 29549163

[eph70375-bib-0030] Laurens, C. , de Glisezinski, I. , Larrouy, D. , Harant, I. , & Moro, C. (2020). Influence of acute and chronic exercise on abdominal fat lipolysis: An update. Frontiers in Physiology, 11, 575363. 10.3389/fphys.2020.575363 PMC7750473 33364972

[eph70375-bib-0031] Li, V. L. , Kim, J. T. , & Long, J. Z. (2020). Adipose tissue lipokines: Recent progress and future directions. Diabetes, 69(12), 2541–2548. 10.2337/dbi20-0012 PMC7679773 33219098

[eph70375-bib-0032] Lønbro, S. , Wiggins, J. M. , Wittenborn, T. , Elming, P. B. , Rice, L. , Pampo, C. , Lee, J. A. , Siemann, D. W. , & Horsman, M. R. (2019). Reliability of blood lactate as a measure of exercise intensity in different strains of mice during forced treadmill running. PLoS ONE, 14(5), e0215584. 10.1371/journal.pone.0215584 PMC6499470 31050686

[eph70375-bib-0033] Many, G. M. , Sanford, J. A. , Sagendorf, T. J. , Hou, Z. , Nigro, P. , Whytock, K. , Amar, D. , Caputo, T. , Gay, N. R. , Gaul, D. A. , Hirshman, M. , Jimenez‐Morales, D. , Lindholm, M. E. , Muehlbauer, M. J. , Vamvini, M. , Bergman, B. , FernáNdez, F. M. , Goodyear, L. J. , Ortlund, E. A. , … MoTrPAC Study Group. (2024). Sexual dimorphism and the multi‐omic response to exercise training in rat subcutaneous white adipose tissue. Nature Metabolism, 6(5), 963–979. 10.1038/s42255-023-00959-9 PMC11132991 38693320

[eph70375-bib-0034] Moraes‐Vieira, P. M. , Saghatelian, A. , & Kahn, B. B. (2016). GLUT4 expression in adipocytes regulates de novo lipogenesis and levels of a novel class of lipids with antidiabetic and anti‐inflammatory effects. Diabetes, 65(7), 1808–1815. 10.2337/db16-0221 PMC4915575 27288004

[eph70375-bib-0035] Mukherjee, S. , Choi, M. J. , Kim, S. W. , & Yun, J. W. (2020). Secreted protein acidic and rich in cysteine (SPARC) regulates thermogenesis in white and brown adipocytes. Molecular and Cellular Endocrinology, 506, 110757. 10.1016/j.mce.2020.110757 32057945

[eph70375-bib-0036] Muscella, A. , Stefàno, E. , Lunetti, P. , Capobianco, L. , & Marsigliante, S. (2020). The regulation of fat metabolism during aerobic exercise. Biomolecules, 10(12), 1699. 10.3390/biom10121699 PMC7767423 33371437

[eph70375-bib-0037] Muthuswamy, K. , Vasanthakumar, K. , Panneerselvan, P. , Thangamani, L. , Krishnan, V. , Piramanayagam, S. , & Subramaniam, S. (2024). FAHFA promotes intracellular calcium signaling via activating the fat taste receptor, CD36 and Src protein kinases in mice taste bud cells. Biochimica et Biophysica Acta (BBA) ‐ General Subjects, 1868(12), 130722. 10.1016/j.bbagen.2024.130722 39426759

[eph70375-bib-0038] Nelson, A. B. , Chow, L. S. , Stagg, D. B. , Gillingham, J. R. , Evans, M. D. , Pan, M. , Hughey, C. C. , Myers, C. L. , Han, X. , Crawford, P. A. , & Puchalska, P. (2022). Acute aerobic exercise reveals that FAHFAs distinguish the metabolomes of overweight and normal‐weight runners. JCI Insight, 7(12), e158037. 10.1172/jci.insight.158037 PMC9057596 35192550

[eph70375-bib-0039] Pajski, M. L. , Maroto, R. , Byrd, C. , & Graber, T. G. (2025). Longitudinal decline of exercise capacity in male and female mice. Journals of Gerontology. Series A, Biological Sciences and Medical Sciences, 80(3), glae293. 10.1093/gerona/glae293 PMC11809237 39693388

[eph70375-bib-0040] Paluchova, V. , Cajka, T. , Durand, T. , Vigor, C. , Dodia, C. , Chatterjee, S. , Fisher, A. B. , & Kuda, O. (2022). The role of peroxiredoxin 6 in biosynthesis of FAHFAs. Free Radical Biology and Medicine, 193, 787–794. 10.1016/j.freeradbiomed.2022.11.015 36403738

[eph70375-bib-0041] Paluchova, V. , Oseeva, M. , Brezinova, M. , Cajka, T. , Bardova, K. , Adamcova, K. , Zacek, P. , Brejchova, K. , Balas, L. , Chodounska, H. , Kudova, E. , Schreiber, R. , Zechner, R. , Durand, T. , Rossmeisl, M. , Abumrad, N. A. , Kopecky, J. , & Kuda, O. (2020). Lipokine 5‐PAHSA is regulated by adipose triglyceride lipase and primes adipocytes for de novo lipogenesis in mice. Diabetes, 69(3), 300–312. 10.2337/db19-0494 PMC7118252 31806624

[eph70375-bib-0042] Pang, Z. , Zhou, G. , Ewald, J. , Chang, L. , Hacariz, O. , Basu, N. , & Xia, J. (2022). Using MetaboAnalyst 5.0 for LC‐HRMS spectra processing, multi‐omics integration and covariate adjustment of global metabolomics data. Nature Protocols, 17(8), 1735–1761. 10.1038/s41596-022-00710-w 35715522

[eph70375-bib-0043] Parsons, W. H. , Kolar, M. J. , Kamat, S. S. , Iii, A. B. , Hulce, J. J. , Saez, E. , Kahn, B. B. , Saghatelian, A. , & Cravatt, B. F. (2016). AIG1 and ADTRP are atypical integral membrane hydrolases that degrade bioactive FAHFAs. Nature Chemical Biology, 12(5), 367–372. 10.1038/nchembio.2051 PMC4837090 27018888

[eph70375-bib-0044] Patel, R. , Santoro, A. , Hofer, P. , Tan, D. , Oberer, M. , Nelson, A. T. , Konduri, S. , Siegel, D. , Zechner, R. , Saghatelian, A. , & Kahn, B. B. (2022). ATGL is a biosynthetic enzyme for fatty acid esters of hydroxy fatty acids. Nature, 606(7916), 968–975. 10.1038/s41586-022-04787-x PMC9242854 35676490

[eph70375-bib-0045] Pavlisova, J. , Bardova, K. , Stankova, B. , Tvrzicka, E. , Kopecky, J. , & Rossmeisl, M. (2016). Corn oil versus lard: Metabolic effects of omega‐3 fatty acids in mice fed obesogenic diets with different fatty acid composition. Biochimie, 124, 150–162. 10.1016/j.biochi.2015.07.001 26151346

[eph70375-bib-0046] Reynaga, I. , Halim, I. A. , & Elbasiouny, S. M. (2025). Sex and age‐related changes in lean mass of C57BL/6 mice: A new method for assessing body composition. Annual International Conference of the IEEE Engineering in Medicine and Biology Society, 2025(1), 1–6. 10.1109/EMBC58623.2025.11254341 PMC12806562 41336696

[eph70375-bib-0047] Riecan, M. , Domanska, V. , Lupu, C. , Patel, M. , Vondrackova, M. , Rossmeisl, M. , Saghatelian, A. , Lupu, F. , & Kuda, O. (2024). Tissue‐specific sex‐dependent difference in the metabolism of fatty acid esters of hydroxy fatty acids. Biochimica et Biophysica Acta (BBA) ‐ Molecular and Cell Biology of Lipids, 1869(8), 159543. 10.1016/j.bbalip.2024.159543 39097081

[eph70375-bib-0048] Riecan, M. , Paluchova, V. , Lopes, M. , Brejchova, K. , & Kuda, O. (2022). Branched and linear fatty acid esters of hydroxy fatty acids (FAHFA) relevant to human health. Pharmacology & Therapeutics, 231, 107972. 10.1016/j.pharmthera.2021.107972 34453998

[eph70375-bib-0049] Romijn, J. A. , Coyle, E. F. , Sidossis, L. S. , Gastaldelli, A. , Horowitz, J. F. , Endert, E. , & Wolfe, R. R. (1993). Regulation of endogenous fat and carbohydrate metabolism in relation to exercise intensity and duration. American Journal of Physiology, 265(3), E380–E391. 10.1152/ajpendo.1993.265.3.E380 8214047

[eph70375-bib-0050] Rossmeisl, M. , Pavlisova, J. , Bardova, K. , Kalendova, V. , Buresova, J. , Kuda, O. , Kroupova, P. , Stankova, B. , Tvrzicka, E. , Fiserova, E. , Horakova, O. , & Kopecky, J. (2020). Increased plasma levels of palmitoleic acid may contribute to beneficial effects of Krill oil on glucose homeostasis in dietary obese mice. Biochimica et Biophysica Acta (BBA) ‐ Molecular and Cell Biology of Lipids, 1865(8), 158732. 10.1016/j.bbalip.2020.158732 32371092

[eph70375-bib-0051] Rossmeislová, L. , Krauzová, E. , Koc, M. , Wilhelm, M. , Šebo, V. , Varaliová, Z. , Šrámková, V. , Schouten, M. , Šedivý, P. , Tůma, P. , Kovář, J. , Langin, D. , Gojda, J. , & Šiklová, M. (2024). Obesity alters adipose tissue response to fasting and refeeding in women: A study on lipolytic and endocrine dynamics and acute insulin resistance. Heliyon, 10(18), e37875. 10.1016/j.heliyon.2024.e37875 PMC11425135 39328508

[eph70375-bib-0052] Ruegsegger, G. N. , & Booth, F. W. (2018). Health benefits of exercise. Cold Spring Harbor Perspectives in Medicine, 8(7), a029694. 10.1101/cshperspect.a029694 PMC6027933 28507196

[eph70375-bib-0053] Sanders, F. W. B. , Acharjee, A. , Walker, C. , Marney, L. , Roberts, L. D. , Imamura, F. , Jenkins, B. , Case, J. , Ray, S. , Virtue, S. , Vidal‐Puig, A. , Kuh, D. , Hardy, R. , Allison, M. , Forouhi, N. , Murray, A. J. , Wareham, N. , Vacca, M. , Koulman, A. , & Griffin, J. L. (2018). Hepatic steatosis risk is partly driven by increased de novo lipogenesis following carbohydrate consumption. Genome Biology, 19(1), 79. 10.1186/s13059-018-1439-8 PMC6009819 29925420

[eph70375-bib-0054] Santoro, A. , Chen, Z. , Nelson, A. T. , Siegel, D. , & Kahn, B. B. (2026). ATGL‐catalyzed biosynthesis mediates the upregulation of Fatty Acid Hydroxy Fatty Acids (FAHFA) levels in white adipose tissue with fasting. Journal of Biological Chemistry, 302(3), 111169. 10.1016/j.jbc.2026.111169 PMC12934275 41570983

[eph70375-bib-0055] Santoro, A. , & Kahn, B. B. (2023). Adipocyte regulation of insulin sensitivity and the risk of Type 2 diabetes. New England Journal of Medicine, 388(22), 2071–2085. 10.1056/NEJMra2216691 37256977

[eph70375-bib-0056] Severinsen, M. C. K. , & Pedersen, B. K. (2020). Muscle‐organ crosstalk: The emerging roles of myokines. Endocrine Reviews, 41(4), 594–609. 10.1210/endrev/bnaa016 PMC7288608 32393961

[eph70375-bib-0057] Sistilli, G. , Kalendova, V. , Cajka, T. , Irodenko, I. , Bardova, K. , Oseeva, M. , Zacek, P. , Kroupova, P. , Horakova, O. , Lackner, K. , Gastaldelli, A. , Kuda, O. , Kopecky, J. , & Rossmeisl, M. (2021). Krill oil supplementation reduces exacerbated hepatic steatosis induced by thermoneutral housing in mice with diet‐induced obesity. Nutrients, 13(2), 437. 10.3390/nu13020437 PMC7912192 33572810

[eph70375-bib-0058] Stanford, K. I. , & Goodyear, L. J. (2016). Exercise regulation of adipose tissue. Adipocyte, 5(2), 153–162. 10.1080/21623945.2016.1191307 PMC4916870 27386159

[eph70375-bib-0059] Stich, V. , De Glisezinski, I. , Crampes, F. , Hejnova, J. , Cottet‐Emard, J.‐M. , Galitzky, J. , Lafontan, M. , Rivière, D. , & Berlan, M. (2000). Activation of alpha(2)‐adrenergic receptors impairs exercise‐induced lipolysis in SCAT of obese subjects. American Journal of Physiology. Regulatory, Integrative and Comparative Physiology, 279(2), R499–R504. 10.1152/ajpregu.2000.279.2.R499 10938238

[eph70375-bib-0060] Thompson, D. , Karpe, F. , Lafontan, M. , & Frayn, K. (2012). Physical activity and exercise in the regulation of human adipose tissue physiology. Physiological Reviews, 92(1), 157–191. 10.1152/physrev.00012.2011 22298655

[eph70375-bib-0061] Tsugawa, H. , Ikeda, K. , Takahashi, M. , Satoh, A. , Mori, Y. , Uchino, H. , Okahashi, N. , Yamada, Y. , Tada, I. , Bonini, P. , Higashi, Y. , Okazaki, Y. , Zhou, Z. , Zhu, Z. J. , Koelmel, J. , Cajka, T. , Fiehn, O. , Saito, K. , Arita, M. , & Arita, M. (2020). A lipidome atlas in MS‐DIAL 4. Nature Biotechnology, 38(10), 1159–1163. 10.1038/s41587-020-0531-2 32541957

[eph70375-bib-0062] West, D. B. , Prinz, W. A. , Francendese, A. A. , & Greenwood, M. R. (1987). Adipocyte blood flow is decreased in obese Zucker rats. American Journal of Physiology, 253(2), R228–R233. 10.1152/ajpregu.1987.253.2.R228 3618823

[eph70375-bib-0063] Yan, S. , Santoro, A. , Niphakis, M. J. , Pinto, A. M. , Jacobs, C. L. , Ahmad, R. , Suciu, R. M. , Fonslow, B. R. , Herbst‐Graham, R. A. , Ngo, N. , Henry, C. L. , Herbst, D. M. , Saghatelian, A. , Kahn, B. B. , & Rosen, E. D. (2024). Inflammation causes insulin resistance in mice via interferon regulatory factor 3 (IRF3)‐mediated reduction in FAHFA levels. Nature Communications, 15(1), 4605. 10.1038/s41467-024-48220-5 PMC11139994 38816388

[eph70375-bib-0064] Yore, M. M. , Syed, I. , Moraes‐Vieira, P. M. , Zhang, T. , Herman, M. A. , Homan, E. A. , Patel, R. T. , Lee, J. , Chen, S. , Peroni, O. D. , Dhaneshwar, A. S. , Hammarstedt, A. , Smith, U. , McGraw, T. E. , Saghatelian, A. , & Kahn, B. B. (2014). Discovery of a class of endogenous mammalian lipids with anti‐diabetic and anti‐inflammatory effects. Cell, 159(2), 318–332. 10.1016/j.cell.2014.09.035 PMC4260972 25303528

[eph70375-bib-0065] Zhou, P. , Santoro, A. , Peroni, O. D. , Nelson, A. T. , Saghatelian, A. , Siegel, D. , & Kahn, B. B. (2019). PAHSAs enhance hepatic and systemic insulin sensitivity through direct and indirect mechanisms. Journal of Clinical Investigation, 129(10), 4138–4150. 10.1172/JCI127092 PMC6763232 31449056

[eph70375-bib-0066] Zhu, Q. F. , Yan, J. W. , Ni, J. , & Feng, Y. Q. (2020). FAHFA footprint in the visceral fat of mice across their lifespan. Biochimica et Biophysica Acta (BBA) ‐ Molecular and Cell Biology of Lipids, 1865(5), 158639. 10.1016/j.bbalip.2020.158639 31988049

